# Assessing Cholesterol Storage in Live Cells and *C. elegans* by Stimulated Raman Scattering Imaging of Phenyl-Diyne Cholesterol

**DOI:** 10.1038/srep07930

**Published:** 2015-01-22

**Authors:** Hyeon Jeong Lee, Wandi Zhang, Delong Zhang, Yang Yang, Bin Liu, Eric L. Barker, Kimberly K. Buhman, Lyudmila V. Slipchenko, Mingji Dai, Ji-Xin Cheng

**Affiliations:** 1 Interdisciplinary Life Science Program, Purdue University, West Lafayette, IN, USA; 2 Department of Comparative Pathobiology, Purdue University, West Lafayette, IN, USA; 3 Department of Chemistry, Purdue University, West Lafayette, IN, USA; 4 National Key Laboratory of Science and Technology on Tunable Laser, Harbin Institute of Technology, Harbin 150080, China; 5 Medicinal Chemistry and Molecular Pharmacology, Purdue University, West Lafayette, IN, USA; 6 Department of Nutrition Science, Purdue University, West Lafayette, IN, USA; 7 Weldon School of Biomedical Engineering, Purdue University, West Lafayette, IN, USA

## Abstract

We report a cholesterol imaging method using rationally synthesized phenyl-diyne cholesterol (PhDY-Chol) and stimulated Raman scattering (SRS) microscope. The phenyl-diyne group is biologically inert and provides a Raman scattering cross section that is 88 times larger than the endogenous C = O stretching mode. SRS microscopy offers an imaging speed that is faster than spontaneous Raman microscopy by three orders of magnitude, and a detection sensitivity of 31 μM PhDY-Chol (~1,800 molecules in the excitation volume). Inside living CHO cells, PhDY-Chol mimics the behavior of cholesterol, including membrane incorporation and esterification. In a cellular model of Niemann-Pick type C disease, PhDY-Chol reflects the lysosomal accumulation of cholesterol, and shows relocation to lipid droplets after HPβCD treatment. In live *C. elegans*, PhDY-Chol mimics cholesterol uptake by intestinal cells and reflects cholesterol storage. Together, our work demonstrates an enabling platform for study of cholesterol storage and trafficking in living cells and vital organisms.

As an important component of cellular membrane, cholesterol controls physical properties of the membrane and contributes to specific membrane structures such as lipid rafts^1^,^2^. Inside cells, cholesterol plays an important role in various signaling pathways^3^,^4^ and serves as the precursor for signaling molecules, and modifies specific proteins, such as hedgehog, to control protein trafficking and activity^5^. The distribution of cholesterol in a living cell is highly regulated^6^,^7^. Intracellular cholesterol is stored in lipid droplets (LDs) in the form of cholesteryl ester to avoid the toxicity caused by free cholesterol^8^,^9^. Dysregulation of cholesterol metabolism and/or trafficking has been linked to diseases, including atherosclerosis^9^,^10^, Niemann-Pick type C (NP-C) disease^11^, and various cancers^12^,^13^. So far, our understanding of cholesterol transport and metabolism is limited, partly due to lack of suitable tools for imaging cholesterol in a living system^14^.

Intracellular cholesterol transport and metabolism have been studied extensively using various reporter molecules^15^, including cholesterol binding molecules and cholesterol analogs. Cholesterol binding molecules, such as cholesterol oxidase, filipin, and perfringolysin O derivatives, are commonly used to study steady-state distribution of cholesterol in fixed cells and tissues^16^,^17^,^18^. Fluorescent cholesterol, including intrinsic fluorescent sterols such as dehydroergosterol (DHE) and fluorophore-tagged analogs such as 7-nitrobenz-2-oxa-1,3-diazole (NBD)-cholesterol and boron dipyrromethene difluoride (BODIPY)-cholesterol are widely used *in vitro* and *in vivo*^19^,^20^,^21^. Radiolabeled cholesterol or its precursors are used in biochemical studies of metabolism and trafficking of cholesterol^15^. More recently, clickable cholesterol analogs were also developed for studying cholesterol-binding proteins and tracking cholesterol metabolism and distribution^22^,^23^.

These current cholesterol assays have limitations. Cholesterol oxidase is commonly used in fluorometric or colorimetric assays to quantify total cholesterol in homogenized cells. Radiolabeled cholesterol has to be used in combination with separation methods to determine intracellular cholesterol distribution indirectly. For imaging purpose, filipin is the most commonly used molecule for visualizing distribution of free cholesterol, but it is only applicable to fixed cells or tissues with moderate specificity because filipin also labels other lipids^24^. BODIPY-cholesterol is known to cause perturbations due to bulkiness of the fluorophores^25^. DHE has the closest structure as cholesterol, but its fluorescence undergoes rapid photo-bleaching^15^, which impedes real-time observation of cholesterol trafficking. Clickable cholesterol analog requires additional steps before fluorescence imaging.

Owing to smaller volumes compared to fluorophores, Raman tags provide a promising way of imaging biomolecules like DNA and cytochrome c inside cells on a Raman microscope^26^,^27^. These Raman tags utilize the vibrational signatures of the carbon-deuterium (C–D) bond, the cyano bond (C≡N) or the alkyne bond (C≡C) that are spectrally isolated from the endogenous Raman bands^28^. Spontaneous Raman imaging has allowed cellular trafficking of different C–D labeled lipid species in fixed cells^29^, and direct visualization of alkyne-tagged DNA synthesis and cytochrome c release from mitochondria in living cells^26^,^27^ with an image acquisition speed of 50 min per frame of 127 × 127 pixels. To enhance the signal level, nonlinear vibrational microscopy based on the coherent Raman process has been developed^30^ and deployed for imaging C–D labeled fatty acids^31^,^32^, amino acids^33^, and drugs^34^ in living cells with a speed that is ~1,000 times faster than with spontaneous Raman microscopy. More recently, stimulated Raman scattering (SRS) imaging of alkyne-tagged molecules has been reported, with a detection limit at the level of hundreds of micromolar^35^,^36^. SRS is a third order nonlinear optical process that involves two laser fields, namely, a pump field at ω_p_ and a Stokes field at ω_S_. When the beating frequency (ω_p_ − ω_S_) is tuned to excite a molecular vibration, the energy difference between ω_p_ and ω_S_ pumps the molecule from a ground state to a vibrationally excited state. In correspondence, the laser fields experience a weak decrease in pump beam intensity, called stimulated Raman loss (SRL), and a corresponding increase in Stokes beam intensity, called stimulated Raman gain (SRG). In the case of SRL, the Stokes beam intensity *I*_S_ is modulated and the pump beam intensity *I*_p_ is recorded by a photodiode. The induced modulation is often extracted by a lock-in amplifier. Theoretically, the modulation depth induced by SRL, *I*_SRL_/*I*_p_, is linearly proportional to the Raman cross section, *σ*, molar concentration of the target molecule, *N*, and the Stokes beam intensity, i.e., *I*_SRL_/*I*_p_ ∝ *σ* *N* *I*_S_. SRS microscopy offers much faster imaging speed compared to spontaneous Raman microscopy^37^.

Here, we report the synthesis of a Raman probe, phenyl-diyne cholesterol (PhDY-Chol), and its use for imaging cholesterol esterification, storage and trafficking inside living cells and vital organisms. By rational design and chemical synthesis, we prepared a probe molecule, PhDY-Chol, which gives a 2,254 cm^−1^ Raman peak that is 88 times stronger than the endogenous C = O stretching band. Compared to alkyne-tagged cholesterol of which the IC_50_ is 16 μM, the phenyl-diyne group is biologically inert and did not cause cytotoxicity after 16 h incubation at 50 μM. In live Chinese hamster ovary (CHO) cells, SRS imaging showed incorporation into plasma membrane, esterification of PhDY-Chol by acyl-CoA: cholesterol acyltransferase 1 (ACAT-1), and storage in LDs. In a cellular model of NP-C disease, PhDY-Chol is selectively accumulated in lysosomes and is esterified and relocated to LDs after treatment with a cholesterol-mobilization drug. In live *C. elegans*, SRS imaging of PhDY-Chol reflected cholesterol uptake through ChUP-1 regulated manner, and storage in the intestinal cells. These studies herald the potential of our method for unveiling intracellular cholesterol trafficking mechanisms and highly efficient screening of drugs that target cholesterol metabolism.

## Results

### Rational design and synthesis of tagged cholesterol with an extremely large Raman scattering cross section

**Figure 1:**
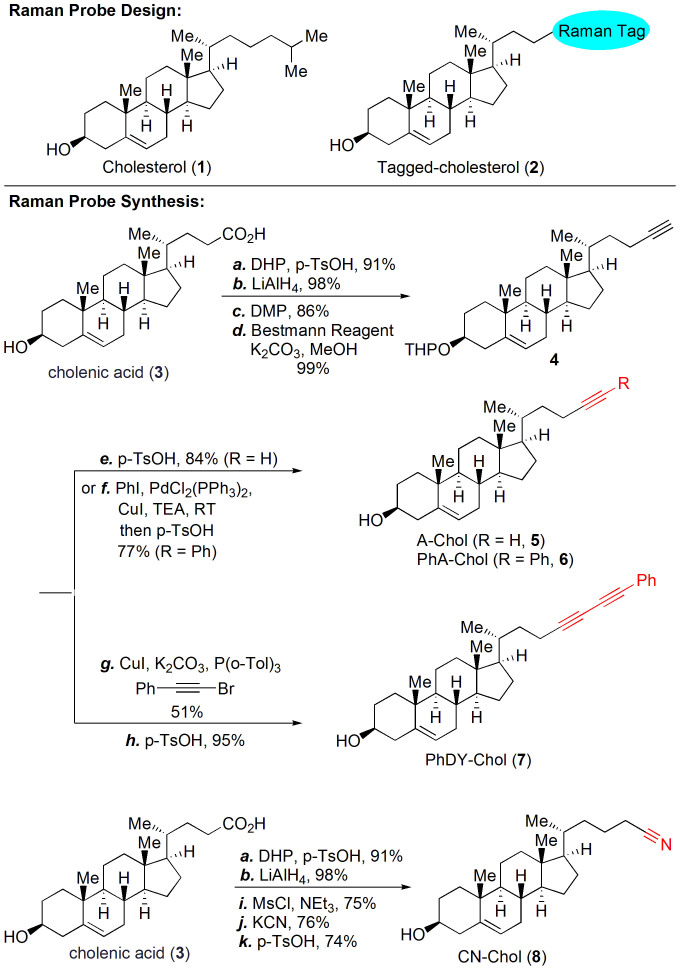
Design and synthesis of tagged cholesterol probes. Reagents and conditions: (a) DHP (5.0 equiv), p-TsOH (0.2 equiv), THF, RT, 91%; (b) LiAlH_4_ (3.0 equiv), THF, 0°C to RT, 98%; (c) DMP (3.0 equiv), NaHCO_3_ (3.0 equiv), CH_2_Cl_2_, 0°C, 86%; (d) dimethyl (1-diazo-2-oxopropyl)phosphonate (Bestmann reagent, 2.4 equiv), K_2_CO_3_ (4.0 equiv), THF/MeOH, RT, 99%; (e) p-TsOH (1.0 equiv), THF/MeOH, RT, 84%; (f) Iodobenzene (1.02 equiv), PdCl_2_(PPh_3_)_2_ (0.05 equiv), CuI (0.05 equiv), TEA, RT; then p-TsOH (1.0 equiv), THF/MeOH, RT, 77%; (g) CuI (0.1 equiv), K_2_CO_3_ (2.0 equiv), P(o-Tol)_3_ (0.2 equiv), phenyl bromoacetylene (1.3 equiv), EtOH, 100°C, 51%; (h) p-TsOH (1.0 equiv), THF/MeOH, RT, 95%; (i) MsCl (3.0 equiv), TEA (3.0 equiv), CH_2_Cl_2_, 0°C to RT, 75%; (j) KCN (2.0 equiv), DMSO, 90°C, 76%; (k) p-TsOH (1.0 equiv), THF/MeOH, RT, 74%. DHP = 3,4-Dihydro-2H-pyran, DMP = Dess-Martin Periodinane, p-TsOH = p-Toluenesulfonic acid, TEA = triethylamine, P(o-Tol)_3_ = tri(o-tolyl)phosphine, MsCl = methanesulfonyl chloride. CN: cyano; A: alkyne; PhA: phenyl-alkyne; PhDY: phenyl-diyne; Chol: cholesterol.

In order to design a probe molecule that not only maintains physiological functions of cholesterol, but also has a large Raman scattering cross section, we chose to replace the aliphatic side chain of cholesterol with a cyano or an alkynyl group (Fig. 1). These groups have small size, which could minimize structural perturbation of the molecule of interest, in this case, cholesterol. Meanwhile, these groups produce strong Raman scattering peaks in a cellular silent region (1,800–2,800 cm^−1^)^26^,^28^ and therefore, can potentially be used for Raman imaging in a low-concentration condition. It has been reported that as the chain length increases, the hyperpolarizability increases in polyynes^38^. Also, aromatic ring capped alkyne was shown to give stronger Raman signals than terminal alkyne^27^. To design tagged cholesterol with very strong Raman intensity, we calculated the Raman cross section of potential tags, namely alkyne, phenyl-alkyne, diyne, and phenyl-diyne, using the Q-Chem and GAMESS electronic structure packages to provide insight of the relation between molecular structure and Raman intensity. Our results show that the localized polarizabilities on each C≡C moiety increase with the number of conjugated triple bonds, as well as with addition of a phenyl ring (Supplementary Figure 1a and Supplementary Table 1). Thus, the total polarizability of the molecule increases as a result of the additive effect as well as non-linear boost in the polarizability of conjugated bonds. The phenyl ring serves as both a donor and an acceptor of π-electrons from the neighboring triple bonds, further escalating polarizabilities of neighboring conjugated bonds. Taking into account that the Raman intensity is proportional to squares of polarizability derivatives, the additional three-fold enhancement of the total polarizability due to conjugation results in a ~10-fold boost in Raman intensity. Together, compared to the alkyne group, the Raman intensity increases by 9 times by adding a phenyl group to the terminal alkyne, and 52 times by conjugating a phenyl group and another alkyne (Supplementary Fig. 1b).

Based on the above considerations, we have synthesized a series of tagged cholesterols – alkyne cholesterol (A-Chol, **5**), phenyl-alkyne cholesterol (PhA-Chol, **6**), phenyl-diyne cholesterol (PhDY-Chol, **7**), and cyano cholesterol (CN-Chol, **8**), as shown in Figure 1. Our synthesis commenced with commercially available cholenic acid **3**. Using a sequence of THP-protection, LiAlH_4_ reduction, Dess-Martin oxidation and Seyferth-Gilbert-Bestmann homologation, cholenic acid **3** was converted to compound **4** with a terminal alkyne group in excellent yield. Removal of the THP-protecting group gave probe **5**. We further prepared PhA-Chol **6** and PhDY-Chol **7** from compound **4** via a palladium-catalyzed Sonogashira reaction and a copper-catalyzed Cadiot-Chodkiewicz reaction, respectively, followed by acidic removal of THP group. Additionally, CN-Chol **8** was prepared from cholenic acid **3** via standard transformations.

### Raman spectral analysis and SRS imaging of tagged cholesterol

**Figure 2:**
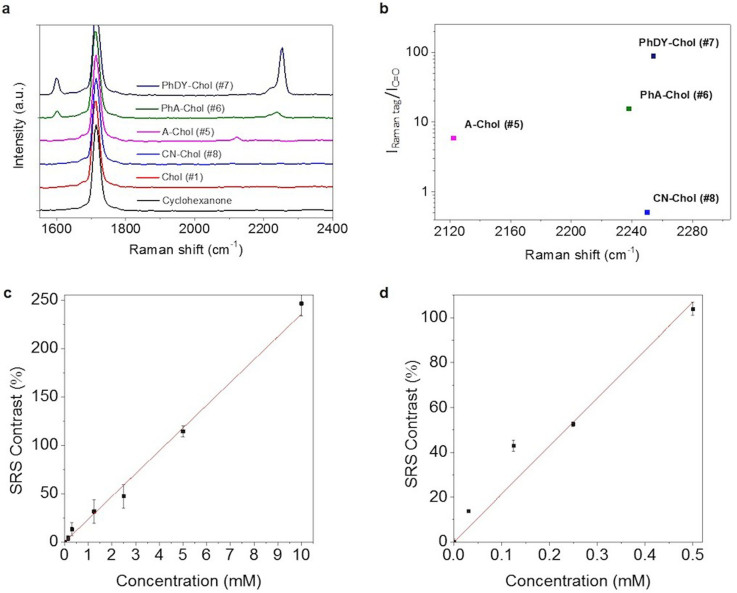
Raman spectral analysis of tagged cholesterol and SRS detection of PhDY-Chol. (a) Raman spectra of 50 mM tagged cholesterols in cyclohexanone (solvent). Spectral intensity was normalized by C = O vibration band at 1,714 cm^−1^. Spectral acquisition time: 10 s. (b) Plot of relative intensity of Raman tags versus solvent and Raman shifts of tagged cholesterols. Based on the molar concentration of the molecules (50 mM) and the solvent (9.7 M), the Raman cross section of C≡C from A-Chol, PhA-Chol, and PhDY-Chol are 6 times, 15 times, and 88 times larger than the C = O band from the solvent, respectively. CN: cyano; A: alkyne; PhA: phenyl-alkyne; PhDY: phenyl-diyne; Chol: cholesterol. (c) SRS contrast versus concentration plot of PhDY-Chol solutions. 13% contrast was reached at 313 μM and 4% contrast was reached at 156 μM. Image acquisition speed: 200 μs per pixel. Data represents the mean ± SEM in 3 measurements. R^2^ = 0.996. (d) SRS contrast versus concentration plot of PhDY-Chol solutions using chirped femtosecond lasers with spectral focusing approach. 14% contrast was reached at 31 μM. Image acquisition speed: 200 μs per pixel. Data represents the mean ± SEM in 3 measurements. R^2^ = 0.980. Contrast was defined as (S − B)/B. S: SRS signal; B: background.

To determine the Raman shift of the C≡C stretching vibrational mode and to compare the level of Raman signals from the tagged cholesterols, we prepared 50 mM of each compound in cyclohexanone and performed confocal Raman spectral analysis (Fig. 2). The signal from CN-Chol was too weak to be detected. A-Chol showed its peak for C≡C vibrational mode at 2,122 cm^−1^; PhA-Chol at 2,239 cm^−1^; PhDY-Chol at 2,254 cm^−1^ (Fig. 2a). To evaluate the amplitude of the Raman scattering cross section, we fitted each Raman band with a Lorentzian profile and calculated the area under the fitted profile. Compared to the Raman peak of each tag to the 1,714 cm^−1^ C = O Raman peak from the solvent (9.7 M for pure cyclohexanone), the alkyne, PhA, and PhDY groups were found to be 6, 15, and 88 times stronger in Raman cross section, respectively (Fig. 2b). This result showed that the PhDY tag produces a spectrally-isolated peak, which is stronger than the C = O vibrational mode by two orders of magnitude.

To determine the SRS imaging sensitivity for PhDY-Chol, we used a femtosecond SRL microscope reported elsewhere^32^. Cyclohexanone solutions of PhDY-Chol were prepared by serial dilution, and SRS images of PhDY-Chol were recorded with the laser beating frequency tuned to be resonant with C≡C vibration at 2,254 cm^−1^. In solutions without PhDY-Chol, a residual background was detected, caused by cross phase modulation. The SRS contrast, defined as (S − B)/B, where S and B denote SRS signal and background, was calculated as a function of PhDY-Chol molar concentration. At the speed of 200 μs per pixel, a linear relationship was observed (Fig. 2c) and 13% and 4% contrasts were reached at 313 μM and 156 μM, respectively. To increase the detection sensitivity, we chirped the femtosecond lasers to 0.8 picoseconds with a SF-10 glass rod. This spectral focusing approach^39^ maintained 85% of the SRS signal while reduced the cross phase modulation background level by 3 times, to a level of 6.3 × 10^−7^ in terms of modulation depth. As a result, the SRS contrast became 14% at 31 μM, corresponding to ~1,800 molecules in the excitation volume (Fig. 2d). We also depicted the modulation depth (ΔI/I) as a function of molar concentration (Supplementary Fig. 2), which is used for estimating the molar concentration of PhDY-Chol inside cells in following studies.

### Cytotoxicity caused by terminal alkyne is avoided by phenyl group

To evaluate the cytotoxicity of tagged cholesterols, we performed MTT cell-viability assays after treating CHO cells with tagged cholesterol. Various concentrations of tagged cholesterol were added to the culture media and the cells were incubated for 48 h before the assays were conducted. A-Chol was found to be toxic to the cells with IC_50_ of 16 μM. Importantly, adding a phenyl group effectively reduced the cytotoxicity (Supplementary Fig. 3a). To directly visualize the toxic effect, we stained the cells with propidium iodide for late apoptosis and necrosis. Cells incubated with A-Chol showed reduced density and extensive apoptosis, whereas both PhA-Chol and PhDY-Chol caused minimum cell death (Supplementary Fig. 3b). This result presents another important role of the phenyl group, which is to reduce the toxicity caused by terminal alkyne. Based on the signal level and the severity of toxicity, we conclude that PhDY-Chol is the most suitable cholesterol analog for live cell imaging, and we used PhDY-Chol in subsequent experiments.

### Membrane incorporation and esterification of PhDY-Chol in live cells

We chose CHO cells which are commonly used for cholesterol trafficking and metabolism studies^40^. To enhance cellular uptake of PhDY-Chol, the cells were pre-incubated in medium supplemented with lipoprotein-deficient serum to deplete medium cholesterol, after which the cells were incubated with 50 μM PhDY-Chol for 16 h. By tuning the laser beating frequency to be resonant with C≡C vibration (2,254 cm^−1^), SRL signals arose from PhDY-Chol. We also tuned the laser to be resonant with C–H vibration (2,885 cm^−1^) and obtained signals from C–H-rich lipid structures, such as LDs.

**Figure 3:**
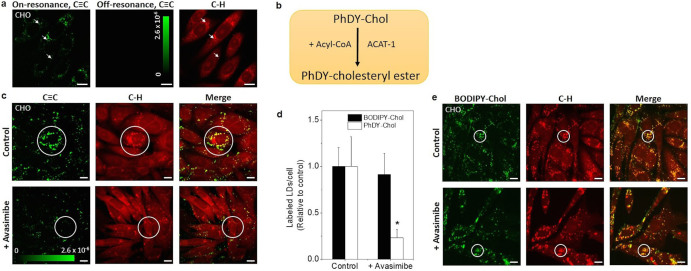
SRS images of PhDY-Chol in live CHO cells and blockage of PhDY-Chol storage into LDs via ACAT-1 inhibition. (a) SRS images of live CHO cells treated with PhDY-Chol (50 μM) for 16 h. C≡C vibrational mode at 2,254 cm^−1^ was used for PhDY-Chol, and C–H vibrational mode at 2,885 cm^−1^ was used for C–H-rich lipid structures. Lasers were also tuned away to 2,099 cm^−1^ to show specificity of PhDY-Chol signal inside the cells. PhDY-Chol was found to accumulate in LDs (arrows). Image acquisition speed: 10 μs per pixel for 512 × 512 pixels. Scalar bar: 10 μm. (b) Schematic graph showing the hypothesis of PhDY-Chol metabolism inside the cells. ACAT-1: Acyl-CoA:cholesterol acyltransferase. (c) SRS images of PhDY-Chol in CHO cells and ACAT-1 inhibited CHO cells by avasimibe treatment. As shown in circles, PhDY-Chol was stored in LDs in CHO cells, but not in avasimibe treated CHO cells. Image acquisition speed: 100 μs per pixel for 400 × 400 pixels. Scalar bar: 10 μm. Intensity bars in (a) and (c) show the ΔI/I of the SRS image. (d) Quantification of PhDY-rich and BODIPY-rich LDs in CHO cells before and after ACAT-1 inhibition. The number of the LDs was normalized by the control group (n = 7). Error bars represent standard deviation. *: p < 0.05. (e) TPEF images of BODIPY-cholesterol and SRS images C–H-rich structures in CHO cells and ACAT-1 inhibited CHO cells. As shown in circles, BODIPY-cholesterol showed no difference between the two groups. Scalar bar: 10 μm.

To show the incorporation of PhDY-Chol into the plasma membrane, we performed spectral focusing SRS imaging of live CHO cells after treating with PhDY-Chol for 1 h with 6 μs per pixel speed. PhDY-Chol in the membrane was detected in the on-resonance image, and the contrast disappeared in the off-resonance image (Supplementary Fig. 4a). The membrane incorporation was confirmed by filipin staining of free cholesterol and Raman spectral analysis (Supplementary Fig. 4b and 4c). By focusing at the filipin-stained membrane, we have obtained the Raman spectrum showing the C = C band from filipin (Supplementary Fig. 4d), the amide I band from protein, and the C≡C band from the PhDY (Supplementary Fig. 4c). Inside live CHO cells, PhDY-Chol was colocalized with LDs found in the C–H vibrational region (Fig. 3a). This colocalization was confirmed by two-photon-excited fluorescence (TPEF) imaging and Raman spectral analysis of BODIPY-stained LDs in fixed CHO cells. (Supplementary Fig. 5a and 5b). The Raman spectra of the BODIPY-labeled LDs showed 702 cm^−1^ peak from cholesterol ring and the C≡C band from the PhDY (Supplementary Fig. 5b), which further supports the localization of PhDY-Chol in LDs. Importantly, high imaging speed offered by SRS microscopy allowed real-time imaging of the trafficking of PhDY-Chol containing LDs within living cells (Supplementary Movie 1).

It is important to note that PhDY-Chol-rich structures inside the CHO cells could not be stained by filipin (Supplementary Fig. 4b), implicating that it is not in the free form. We hypothesize that PhDY-Chol is converted into PhDY-cholesteryl ester, by ACAT-1, the enzyme responsible for cholesterol esterification^8^ (Fig. 3b). To confirm the esterification of PhDY-Chol, we inhibited ACAT with avasimibe for 24 h before addition of PhDY-Chol. After blocking cholesterol esterification, the amount of PhDY-Chol storage found in CHO cells significantly decreased (Fig. 3c). Although LDs were still visible, the amount of PhDY-Chol signal found inside LDs reduced by 4 times (Fig. 3d). ACAT-1 knockdown by shRNA was also conducted to specifically inhibit the enzyme. Similarly, we found decreased amount of PhDY-Chol in ACAT-1 knocked down CHO cells, and the amount of PhDY-Chol in LDs reduced significantly (Supplementary Fig. 6a). To determine where PhDY-Chol accumulates after ACAT-1 inhibition, we stained the cells with LysoTracker for lysosomes. Our result indicated that after ACAT-1 inhibition, PhDY-Chol partially located in lysosomes (Supplementary Fig. 6b). Collectively, these results show that PhDY-Chol can be transported into cells, converted into PhDY-cholesteryl ester by ACAT-1, and stored in LDs following the normal metabolic pathway of cholesterol. To emphasize the physiological compatibility of our PhDY tag, we treated CHO cells with BODIPY-cholesterol. The amount of BODIPY-cholesterol incorporated into LDs did not change after ACAT-1 inhibition (Fig. 3d and 3e), indicating that BODIPY-cholesterol directly labels the LDs without metabolic conversion into cholesteryl ester. It is known that excess cholesterol inside cells is stored into LDs through cholesterol esterification by ACAT proteins^8^. Therefore, PhDY-Chol reflects the intracellular cholesterol metabolism more faithfully compared to BODIPY-cholesterol.

### Lysosomal accumulation and relocation to LDs in NP-C disease model

**Figure 4:**
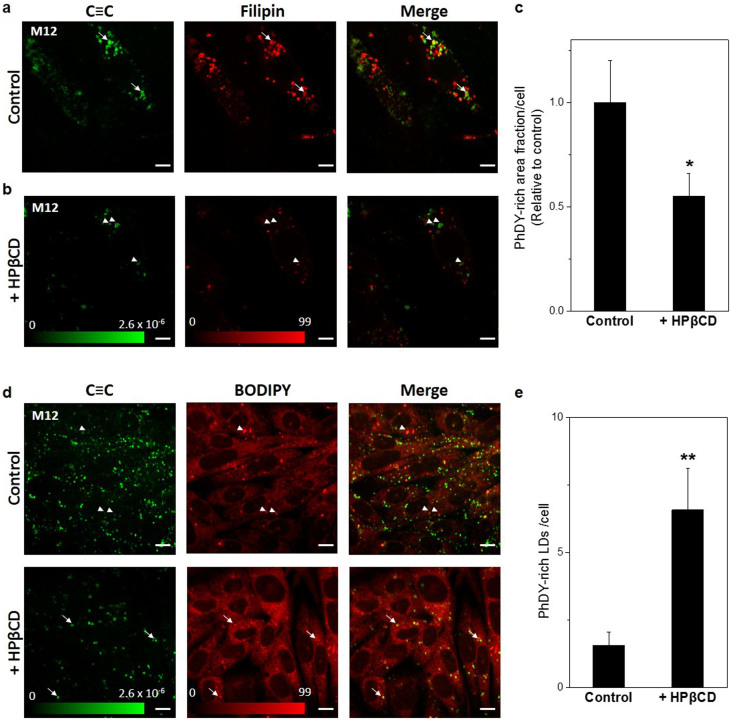
Restored cholesterol transport in M12 cells treated with HPβCD. TPEF images of filipin and SRS images PhDY-Chol in (a) PhDY-Chol-loaded M12 cells, and (b) the same cells treated with HPβCD (500 μM) for 30 h. Arrows indicate PhDY-rich area labeled by filipin before treatment (non-esterified PhDY-Chol), and arrow heads indicate PhDY-rich area not labeled by filipin after treatment (esterified PhDY-Chol). Green intensity bar shows the ΔI/I value of the SRS image; red intensity bar represents the relative intensity of fluorescence. Image acquisition speed: 100 μs per pixel for 400 × 400 pixels. Scalar bar: 10 μm. (c) Quantification of PhDY-rich area in the cells before and after HPβCD treatment (n = 7). Error bars represent standard deviation. *: p < 0.05. (d) TPEF images of BODIPY and SRS images of PhDY-Chol in M12 cells treated with or without HPβCD (500 μM) for 30 h. Arrow heads indicate LDs without PhDY-Chol before treatment, and arrows indicate LDs with PhDY-Chol after treatment. Green intensity bar shows the ΔI/I value of the SRS image; red intensity bar represents the relative intensity of fluorescence. Image acquisition speed: 100 μs per pixel for 400 × 400 pixels. Scalar bar: 10 μm. (e) Quantification of PhDY-rich LDs in the cells before and after HPβCD treatment (n = 7). Error bars represent standard deviation. **: p < 0.005.

Next, we explored the potential of PhDY-Chol for studying cholesterol transport in NP-C disease, a disorder featured by abnormal cholesterol accumulation in late endosome/lysosome caused by mutation in *NPC1* or *2* gene^41^. M12 cells, mutant CHO cells that contain a deletion of the *NPC1* locus, were established as a cellular model of the NP-C disease^42^. By combining SRL imaging of PhDY with TPEF imaging of filipin, we observed that, unlike wildtype CHO cells, the PhDY-Chol-rich structures were stained by filipin, indicating that these PhDY-Chol molecules were located in lysosomes. (Fig. 4a, Supplementary Fig. 7a and 7b). Moreover, we observed some filipin labeled structures that do not contain PhDY-Chol. This result is reasonable given that filipin has been shown to label other lipid molecules, such as glycosphingolipids^24^. As additional evidence, we incubated M12 cells with PhDY-Chol and stained the cells with LysoTracker. It was found that all PhDY-Chol-rich areas were localized in LysoTracker-stained organelles (Supplementary Fig. 7c). Collectively, these results showed that PhDY-Chol selectively represents the lysosomal storage of cholesterol in the NP-C disease model.

We then treated the PhDY-Chol-labeled M12 cells with a cholesterol-mobilizing drug, hydroxypropyl β-cyclodextrin (HPβCD)^43^. This drug is known to mediate lysosomal escape of cholesterol, and promote storage of excess cholesterol into LDs^44^. After treating with HPβCD, the amount of PhDY-Chol in M12 cells decreased by half (Fig. 4b and c). Interestingly, we observed that some PhDY-Chol-rich areas were not labeled by filipin after HPβCD treatment (arrow heads in Fig. 4b). These areas likely represent PhDY-cholesteryl ester stored in LDs. To confirm this possibility, we stained the cells with BODIPY for localization of LDs. The result clearly showed that PhDY-Chol has moved into LDs after HPβCD treatment, and the number of PhDY-rich LDs increased significantly (Fig. 4d and 4e). Together, these data indicate that PhDY-Chol can be used as a reliable cholesterol analog to study cholesterol mobilization inside living cells.

### Cholesterol uptake and storage in intestinal cells in *C. elegans* visualized by PhDY-Chol

**Figure 5:**
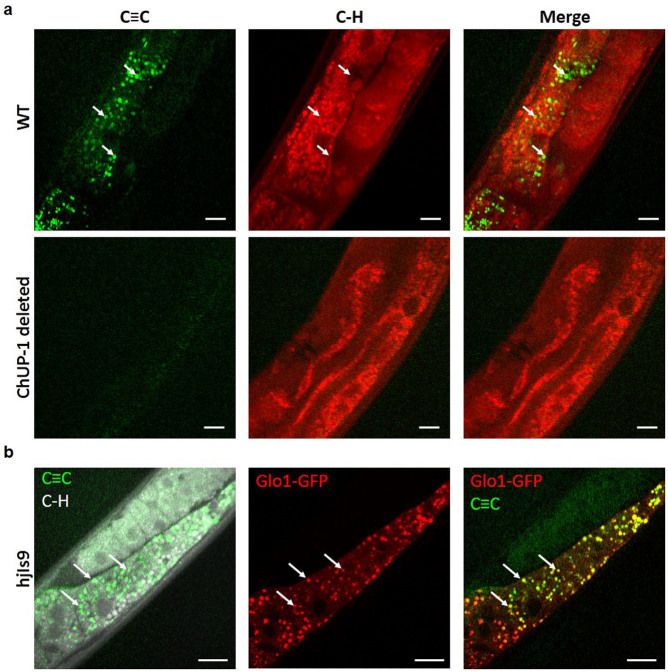
SRS imaging of PhDY-Chol visualizes compartments of cholesterol storage in live *C. elegans*. (a) SRS images of live wildtype and ChUP-1 deleted *C. elegans* fed with PhDY-Chol (500 μM) for 3 days. Arrows indicate PhDY-rich particles in the intestine. Image acquisition speed: 40 μs per pixel for 400 × 400 pixels. Scalar bar: 10 μm. (b) TPEF and SRS images of live hjIs9 [ges-1p::glo-1::GFP + unc-119(+)] worm fed with PhDY-Chol (500 μM) for 3 days. Arrows indicate the PhDY-rich particles in LROs. Image acquisition speed: 40 μs per pixel for 400 × 400 pixels. Scalar bar: 10 μm.

Finally, to demonstrate the capability of monitoring cholesterol uptake and distribution *in vivo*, we used *C. elegans* as an animal model to study cholesterol uptake and storage. We fed N2 wildtype *C. elegans* with PhDY-Chol-labeled *E. coli* and imaged PhDY-Chol storage in the worms using our SRL microscope at speed of 40 μs per pixel. PhDY-Chol was found most abundantly in the intestinal cells inside the wildtype worms (upper panels in Fig. 5a). To confirm the uptake of PhDY-Chol by intestinal cells, we fed ChUP-1 mutant *C. elegans*, in which dietary cholesterol uptake is inhibited by ChUP-1 deletion^45^, with PhDY-Chol. We did not observe PhDY-Chol inside this strain (lower panels in Fig. 5a), which indicates that the PhDY tag did not affect the cholesterol uptake process. Then, we tuned the laser to be resonant with C–H vibration for lipid-rich LDs. Unlike CHO cells, the PhDY-Chol-rich compartments were found to be distinguished from LDs in wildtype worms (upper panels in Fig. 5a). To explore the nature of PhDY-Chol-rich compartments found in our study, we used hjIs9 worms that contain GFP targeted to lysosome-related organelles (LROs) in intestinal cells^46^. Dual-modality SRS and TPEF imaging showed that PhDY-Chol is stored in the LROs (Fig. 5b). We further confirmed the cholesterol storage in intestinal LROs by combining TPEF imaging of GFP targeted LROs and Raman spectral analysis of hjIs9 worms fed with normal cholesterol (Supplementary Fig. 8). Raman spectrum of LROs showed peaks for sterol C = C bond at 1,667 cm^−1^ and Fermi resonance between asymmetrical CH_2_ vibrational modes at 2,875 cm^−1^, indicating the presence of cholesterol in this organelle. Collectively, these results suggest that dietary PhDY-Chol uptake is through a ChUP-1 mediated process, and unlike mammalian CHO cells, *C. elegans* stores cholesterol in LROs, but not in LDs in the intestine.

## Discussion

In this study, we have developed a series of tagged cholesterols based on quantum chemistry calculations and chemical synthesis. By using PhDY to replace the aliphatic chain in cholesterol, we produced a cholesterol analog, PhDY-Chol, with a Raman signal that is two orders of magnitude stronger than the C = O group. By SRS imaging of live CHO cells, PhDY-Chol was found to be incorporated into the membrane, and converted to PhDY-cholesteryl ester for storage in LDs. With this cholesterol analog, we experimentally validated that after ACAT-1 inhibition, cholesterol partly accumulates in lysosomes. In live *NPC1*-deleted CHO cells, PhDY-Chol selectively represented lysosomal accumulation of cholesterol in untreated cells, and esterification and relocation to LDs after HPβCD treatment. Lastly, SRS imaging of PhDY-Chol in live *C. elegans* showed cholesterol uptake by intestinal cells, and indicated LROs, but not LDs, as the cholesterol storage compartments in the intestine.

Essential parameters of a valid Raman tag include its amplitude of Raman scattering cross section, cytotoxicity, and biocompatibility. Although the C–D bond can be used to replace C–H bonds without changing the structures of the molecules, it gives relatively weak Raman intensities. Raman signal from alkyne bond is stronger than that from C–D bond by one order of magnitude^27^, and detection at hundreds of micromolar of alkyne-containing molecules by SRS microscopy was reported^35^,^36^. It should be noted that terminal alkyne is known to react with the cysteine residues in proteins^47^, which might induce cytotoxicity at micromolar concentrations. In our study, through rational design and synthesis of a PhDY tag, we increased the Raman scattering cross section by 15 times compared to the alkyne group, and 88 times compared to the endogenous C = O group. This enhancement is a result of conjugation of π-electrons among the two C≡C bonds and the phenyl group. As a result, we have been able to detect ~30 μM of PhDY-Chol molecules (~1,800 molecules at excitation volume), and demonstrated SRS imaging of PhDY-Chol in single membrane at speed of 6 μs per pixel, and a real-time movie of PhDY-Chol containing LDs. Importantly, this design also shielded the activity of terminal alkyne and significantly reduced cytotoxicity. Moreover, PhDY-Chol structurally mimics cholesterol, using the same physiological process for cholesterol transport and metabolism inside cells. Cell membrane morphology did not alter when PhDY-Chol was supplemented at high concentrations when compared to the cells supplemented with the same concentrations of cholesterol (Supplementary Fig. 3). Moreover, the fluorescent property of pyrenedecanoic acid, a membrane fluidity indicator^48^, did not change after CHO cells were treated with 50 μM of PhDY-Chol or 50 μM of cholesterol for 16 h. These evaluations suggest that the membrane property of the cells was not significantly affected by addition of PhDY-Chol or cholesterol under our experimental conditions. We note that using the same strategy, other Raman tag molecules can be designed for sensitive and biocompatible probing of biomolecules in live cells.

The potential value of a Raman tag is also related to the detection sensitivity of SRS microscopy. One limitation comes from the cross phase modulation, which produces a background that reduces the contrast for the tag molecules. Although broadband femtosecond lasers provide high peak intensity to enhance the SRS signal^32^, they also increase the amplitude of the cross phase modulation. As shown in our study, this background can be reduced by 3 times using spectral focusing^39^. The spectral focusing approach also increases the spectral selectivity, reduces the photodamage, and provides opportunities to conduct hyperspectral SRS imaging^49^.

In this study, we compared BODIPY-cholesterol^21^ and PhDY-Chol. Our results show that PhDY-Chol is stored in LDs via esterification which can be blocked by ACAT-1 inhibition. In contrast, BODIPY-cholesterol labels LDs even after ACAT-1 inhibition. This result may be due to the strong hydrophobic interaction of BODIPY with LDs, and is consistent with previous studies showing that BODIPY-cholesterol is hardly esterified by ACAT-1 inside the cells^21^. These results demonstrate that PhDY-Chol, but not BODIPY-cholesterol, reflects the intracellular behavior of free cholesterol. We also showed that PhDY-Chol reflects the location of the cholesterol in real-time, unlike filipin staining, which requires fixation. Lastly, SRS microscopy utilizes chemical-bond vibrational signals for visualization. Thus, unlike fluorophores, the PhDY tag does not undergo bleaching (Supplementary Fig. 9), in contrast to BODIPY-cholesterol and DHE^15^ which is known to have a rapid photo-bleaching rate. Combining these unique properties, PhDY-Chol allows quantitative imaging of intracellular cholesterol, and repetitive observation of the same sample before and after treatment.

Our work opens new opportunities for mechanistic study of the NP-C disease, a fatal neurodegenerative disease that shows extensive lysosomal accumulation of cholesterol. Early detection methods and treatment strategies of this disease are still under development^11^. The involvement of lysosomal cholesterol accumulation to the neurodegeneration is still unclear^50^. Our *in vitro* study shows the cholesterol trafficking and metabolism in a cellular model. This can be extended to *in vivo* studies using suitable mouse models to understand the progression of the disease and impact of potential therapeutic strategies, especially in central nervous system.

*C. elegans* is an important model for genetic and chemical screening in many diseases^51^. It has been proposed that the intracellular sterol trafficking pathway might be conserved in nematodes^52^, making it a good model for exploring the genetics of fat storage and lipid metabolism^53^. However, lipid storage in *C. elegans* has been a debate because of limitations and controversies of the visualization tool for lipids^54^,^55^, especially cholesterol. Filipin labeling causes sterol extraction and some tissues were not accessible with staining^20^,^56^. Imaging fluorescent cholesterol in *C. elegans* is a challenge due to strong and spectrally overlapping autofluorescence from the worm^57^. Using label-free coherent anti-Stokes Raman scattering (CARS) microscopy, fat storage compartments in *C. elegans* were studied^58^,^59^. However, single color CARS microscopy based on the signal from C–H stretching vibrations cannot tell the compositions of the LDs, and so far it is not clear where the cholesterol is stored inside the worms. By combination of chemical synthesis of PhDY-Chol and real-time SRS imaging, we found evidences suggesting the cholesterol storage in LROs. Label-free Raman spectral analysis was performed to validate the finding. Sterol uptake and transport in worms are still poorly understood^45^, and SRS imaging of PhDY-Chol opens an avenue to directly assess cholesterol uptake and transport for genome-wide RNA interference screening of cholesterol transport and storage genes in this animal model. Finally, our work also opens new opportunities to study cholesterol trafficking and metabolism in other animal models such as zebrafish and mice.

## Methods

### Calculation of Raman intensity

All calculations were performed at the HF/6-311G* level of theory. Geometry optimizations, vibrational frequencies and Raman intensities are obtained in Q-Chem electronic structure package^60^. Localized polarizabilities are calculated in the GAMESS quantum chemistry software^61^.

### Chemicals

3β-hydroxy-Δ^5^-cholenic acid was purchased from VWR. Lipoprotein-deficient serum was purchased from Biomedical Technologies Inc. Cholesterol, avasimibe, and filipin complex were purchased from Sigma-Aldrich. BODIPY-cholesterol was purchased from Avanti Polar Lipids, Inc. Propidium iodide, BODIPY, and LysoTracker were purchased from Life technologies.

### Synthesis of Raman-tagged cholesterol

Detailed synthesis procedures and characterization of compounds are shown in Supplementary Information.

### Solubilization of tagged cholesterol

To solubilize the tagged cholesterol molecules, the following procedure was used to prepare a stock solution of 10 mM cholesterol probe molecules. The appropriate amount of cholesterol probe powder was dissolved in 100% ethanol to make a 20 mM solution. The tube was vortexed and then sonicated in bath sonicator for 2 min. The same volume of DMSO was added into the tube, vortexed, and then sonicated in bath sonicator for 2 min. BODIPY-cholesterol was prepared with the same procedure. For SRS imaging and Raman spectral analysis of tagged cholesterol solutions, cholesterol probe molecules were prepared in cyclohexanone at 50 mM. The tube was vortexed and then sonicated in bath sonicator for 2 min. 1 μL of the solution was taken to prepare cover glass samples immediately before use.

### Cell culture and PhDY-Chol treatment

CHO-K1 cells and M12 cells (mutant CHO-K1 cells that contain a deletion of the NPC1 locus^42^) were kindly provided by Dr. Daniel Ory, and were grown in a monolayer at 37°C in 5% CO_2_ in DMEM/F-12 medium supplemented with 10% (vol/vol) FBS. To incubate cells with PhDY-Chol, cells were pre-incubated in DMEM/F-12 medium supplemented with 4.4% lipoprotein-deficient serum to deplete medium cholesterol for 16 h. The cells were then incubated with PhDY-Chol containing medium (DMEM/F-12 + 4.4% lipoprotein-deficient serum +50 μM PhDY-Chol) for 16 h to 24 h. Cells were rinsed with 1× PBS buffer three times before the next procedure.

### Raman spectromicroscopy

The background of Raman spectrum was removed as described^62^. Each Raman spectrum of tagged cholesterol solution was acquired in 10 seconds, and each Raman spectrum of fluorescence stained cells was acquired in 30 seconds. On the same microscope, TPEF imaging was performed with 707 nm laser with 100 mW power. Backward-detected two-photon fluorescence signal was collected through a 425/40 nm or 522/40 nm band-pass filter for imaging filipin or BODIPY fluorescence, respectively.

### SRS microscopy

SRS imaging was performed on a femtosecond SRL microscope, with the laser beating frequency tuned to the C≡C vibration band at 2,254 cm^−1^, or to the C–H vibration band at 2,885 cm^−1^, as described previously^32^. The laser power at the specimen was maintained at 75 mW, and no cell or tissue damage was observed. For off-resonance, 2,099 cm^−1^ was used. On the same microscope, TPEF imaging was performed with 843 nm laser with 30 mW power. Forward-detected two-photon fluorescence signal was collected through an appropriate band-pass filter for imaging filipin, BODIPY, or LysoTracker.

### ACAT-1 inhibition

ACAT-1 inhibition was used to block cholesterol esterification either by adding a potent ACAT inhibitor, avasimibe, or by RNA interference with ACAT-1 shRNA plasmid. Avasimibe: Cells were pre-treated with avasimibe at a final concentration of 10 μM for 24 h. Then PhDY-Chol containing medium with 10 μM avasimibe was added into the cells and incubated for 24 h. RNA interference: RNA interference was employed to specifically inhibit endogenous ACAT-1. The ACAT-1 shRNA plasmid was purchased from Santa Cruz (sc-29624-SH). shRNA plasmid was transfected with Lipofectamine®2000 (Invitrogen 11668030) as described in the manufacturer's protocols.

### HPβCD treatment

HPβCD was used as a drug treatment of NP-C disease. M12 cells were incubated with PhDY-Chol for 16 h as described above. Then cells were treated with 500 μM HPβCD for 30 h.

### Cell-viability assay

CHO cells were grown in 96-well plates with density of 4000 cells per well. The next day, the cells were treated with each cholesterol probe at the indicated concentrations for 48 h. Cell-viability was measured with the MTT colorimetric assay (Sigma).

### Propidium iodide staining

Propidium iodide was used to stain late apoptotic or necrotic cells. CHO cells were incubated with 30 μM of tagged cholesterol molecules for 24 h. The propidium iodide staining was performed following protocols provided by the manufacturer (Life Technologies).

### Fluorescent staining of free cholesterol, LDs, and lysosomes

Filipin was used to stain free cholesterol. Cells were fixed with 10% formalin solution (Sigma) for 1 h at room temperature. 1.5 mg/mL glycine in PBS was used to quench the formalin by incubating the fixed cells for 10 minutes at room temperature. To stain the cells with filipin, working solution of 0.05 mg/mL of filipin in PBS/10% FBS was used to incubate cells for 2 h at room temperature. BODIPY was used to label LDs. Cells were incubated with 10 μg/mL of BODIPY for 30 minutes at room temperature. LysoTracker Yellow-HCK-123 was used to stain lysosomes following protocols provided by the manufacturer (Life Technologies). Cells were rinsed with PBS three times before TPEF imaging.

### *C. elegans* strains

The N2 Bristol was used as wild-type strain. VC452 strain with chup-1(gk245) X genotype was used to study PhDY-Chol uptake. VS17 strain with hjIs9 [ges-1p::glo-1::GFP + unc-119(+)] genotype was used to study cholesterol storage in LROs.

### PhDY-Chol uptake into *C. elegans*

PhDY-Chol uptake procedure was modified from a previously reported procedure^20^. Briefly, 500 μM of PhDY-Chol in DMSO was spread on the NGM plates seeded with an *E. coli* OP50 lawn and allowed to grow overnight at room temperature. *C. elegans* was then transferred to PhDY-Chol containing plates and grown for 3 days before SRS imaging.

### Statistical analysis

To quantify PhDY-rich area, we first selected one cell and used “Threshold” function to select PhDY-rich cellular regions using ImageJ. Then, by using “Analyze Particles” function, the area fraction (%) of PhDY-rich region was obtained. To quantify PhDY-rich LDs, “Image Calculator” function in ImageJ was used to multiply SRS image of PhDY-Chol by TPEF image of BODIPY. Then, after using “Threshold” function to select PhDY-rich LDs, the number of PhDY-rich LDs was counted by “Analyze Particles” function. For each group, 7 cells were analyzed, and results were shown as mean ± standard deviation (SD). Student's t test was used for all the comparisons. p < 0.05 was considered statistically significant.

## Author Contributions

M.D. and J.X.C. designed the research. D.Z. and L.V.S. performed and analyzed the quantum chemistry calculation. W.Z. and Y.Y. synthesized and analyzed the compounds. H.J.L. and D.Z. performed and analyzed the SRS experiments. B.L. developed dual-modality imaging. E.L.B. and K.K.B. provided the NP-C disease model and discussed the data. H.J.L. performed and analyzed *in vitro* and *in vivo* experiments. H.J.L., W.Z., M.D. and J.X.C. wrote the manuscript.

## Supplementary Material

Supplementary Information Supplementary Movie

Supplementary Information Supplementary Information
