# Supplementary material for: Assessing Cholesterol Storage in Live Cells and C. elegans by Stimulated Raman Scattering Imaging of Phenyl-Diyne Cholesterol
Source: Sci Rep. 2015 Jan 22;5:7930. doi: 10.1038/srep07930 (PMC4302291; doi:10.1038/srep07930)
Supplement: Supplementary Information [file srep07930-s2.pdf]

Supplementary Information to:

Assessing Cholesterol Storage in Live Cells and *C. elegans* by Stimulated Raman Scattering Imaging of Phenyl-Diyne Cholesterol

Hyeon Jeong Lee, Wandi Zhang, Delong Zhang, Yang Yang, Bin Liu, Eric L. Barker, Kimberly K. Buhman, Lyudmila V. Slipchenko, Mingji Dai*, Ji-Xin Cheng*

Content

- **Supplementary Results– Page 2 – 11**
- **Synthesis of Probes – Page 12 – 19**
- **Supplementary References – Page 20**
- **NMR Spectra of New Compounds – Page 21 – 32**

Supplementary Results

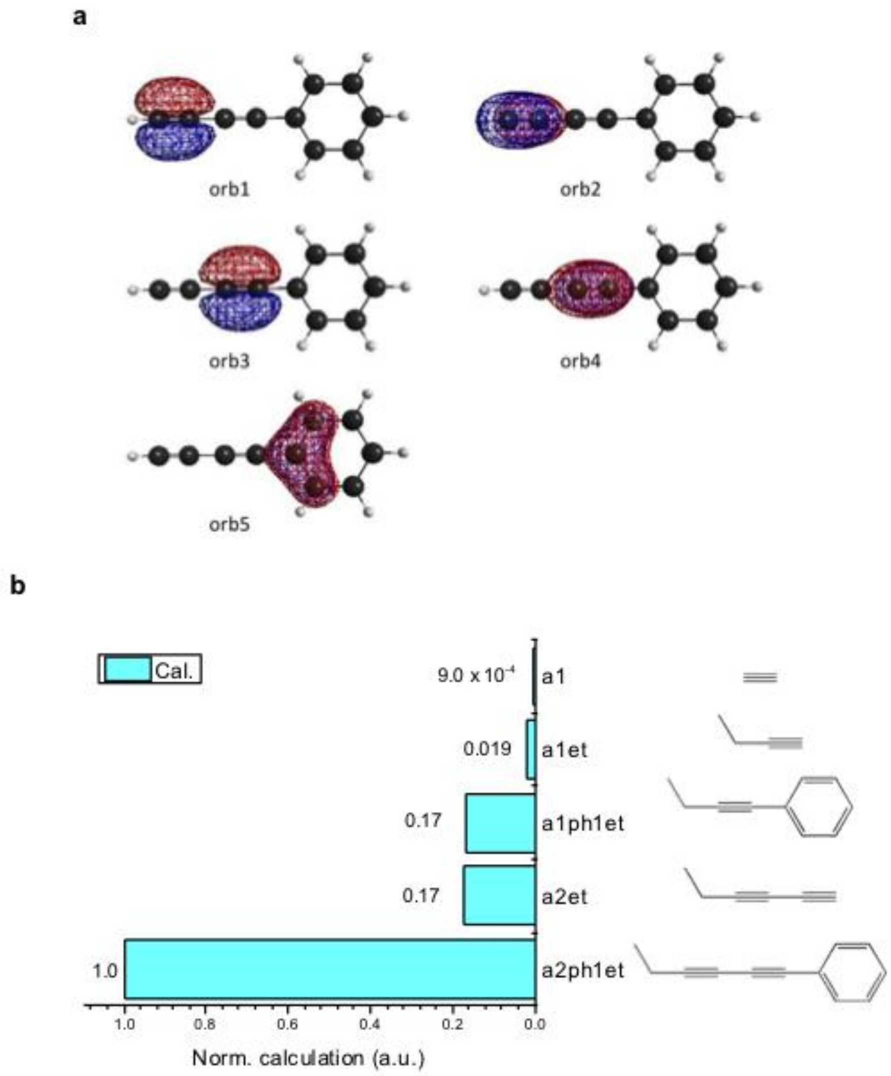

Supplementary Figure 1. Theoretical Raman intensities of the C≡C stretching mode in various tags. The total molecular polarizability is broken down in terms of the value of the polarizability corresponding to each bond in the molecule. The Raman scattering cross section arises from the polarizability caused by conjugation of the π-electrons of the alkyne and the phenyl groups. The π-orbitals possess large polarizability tensors, such that the polarizability of the triple bond is mainly determined by the polarizability of π-orbitals. **(a)** Depiction of symmetry-localized π-orbitals in C_4H-C_6H_5 (C≡C-C≡C-Ph). The distributed polarizabilities corresponding to the localized orbitals on C≡C and phenyl ring are shown in Supplementary Table 1, such that the polarizabilities of orbitals 1 and 2 determine the total polarizability of the left C≡C bond, polarizabilities of orbitals 3 and 4 sum up to the polarizability of the middle C≡C bond. Additionally, the closest to alkyne part of the ring (orbital 5) exhibits significant change in polarizability along triple-bond stretching vibration, and therefore polarizabilities corresponding to this orbital are included in the total count of polarizability of the triple-bond system. Distributed polarizabilities are calculated using GAMESS electronic structure package[1]. **(b)** Comparison of C≡C stretching Raman intensities in various tags computed using the Q-Chem quantum chemistry software[2]. Intensities were normalized to the highest intensity.

Supplementary Table 1. Distributed polarizabilities of C≡C and phenyl moieties calculated as sums of polarizabilities of localized π-orbitals in various tags[b]

	XX[a]	average
**C≡C**	**2.82**	**1.60**
**C≡C-Ph**	**7.42**	**3.76**
C≡C	4.62	2.15
Ph	2.80	1.61
**C≡C-C≡C**	**8.70**	**3.54**
C≡C	4.35	1.77
C≡C	4.35	1.77
**C≡C-C≡C-Ph**	**16.70**	**7.37**
left C≡C	5.77	2.51
middle C≡C	7.20	2.95
Ph	3.73	1.91

[a] XX: the largest components
[b] The total polarizability of π-conjugated system increases as a result of the additive effect as well as non-linear boost in the polarizability of conjugated bonds.

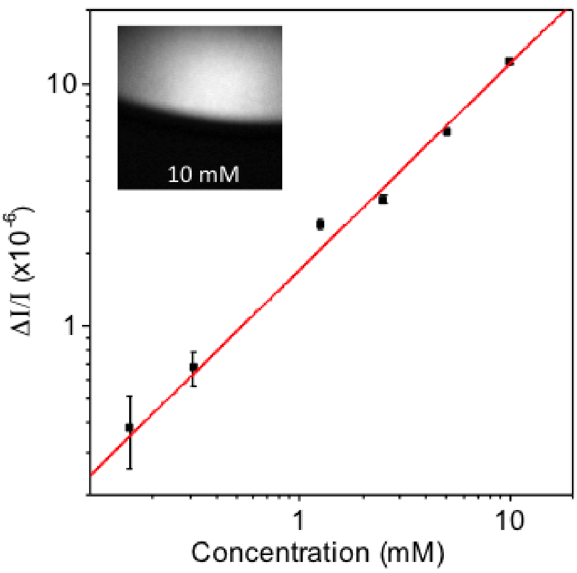

Supplementary Figure 2. Linear correlation between PhDY-Chol concentration and modulation depth. Concentration of PhDY-Chol and modulation depth (ΔI/I) show linear correlation, which can be expressed as: y = 0.85x – 5.77 (R^2 = 0.98). Inset is an SRS image of 10 mM PhDY-Chol in cyclohexanone. Data acquisition speed: 200 μs per pixel. Error bars represent standard errors.

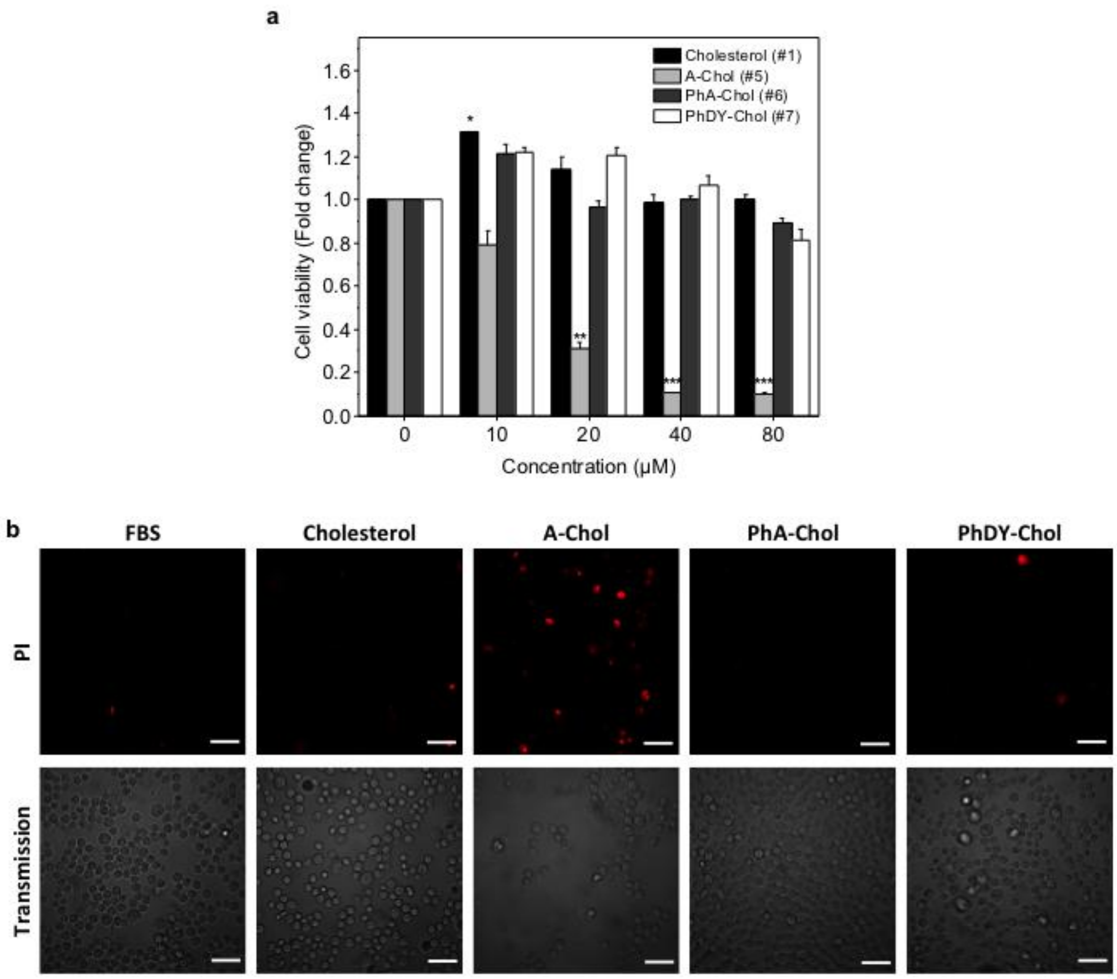

Supplementary Figure 3. Phenyl group prevented cytotoxicity of the probe molecules. (a) MTT Cell-viability assays show that A-Chol is toxic to the cells, but phenyl group prevents the cytotoxicity. CHO cells were incubated with each probe in various concentrations for 48 h before MTT cell-viability assays were conducted. Error bars represent standard error of the mean (SEM). $n > 3$, *: $p < 0.05$; **: $p < 0.005$; ***: $p < 0.0005$. **(b)** Propidium iodide staining shows that A-Chol induces apoptosis and necrosis. Transmission images show reduced cell number in A-Chol treated CHO cells. Phenyl group prevented the cytotoxic effect. A-Chol: alkyne cholesterol; PhA-Chol: phenyl-alkyne cholesterol; PhDY-Chol: phenyl-diyne cholesterol; PI: propidium iodide. Scale bar: 50 μm.

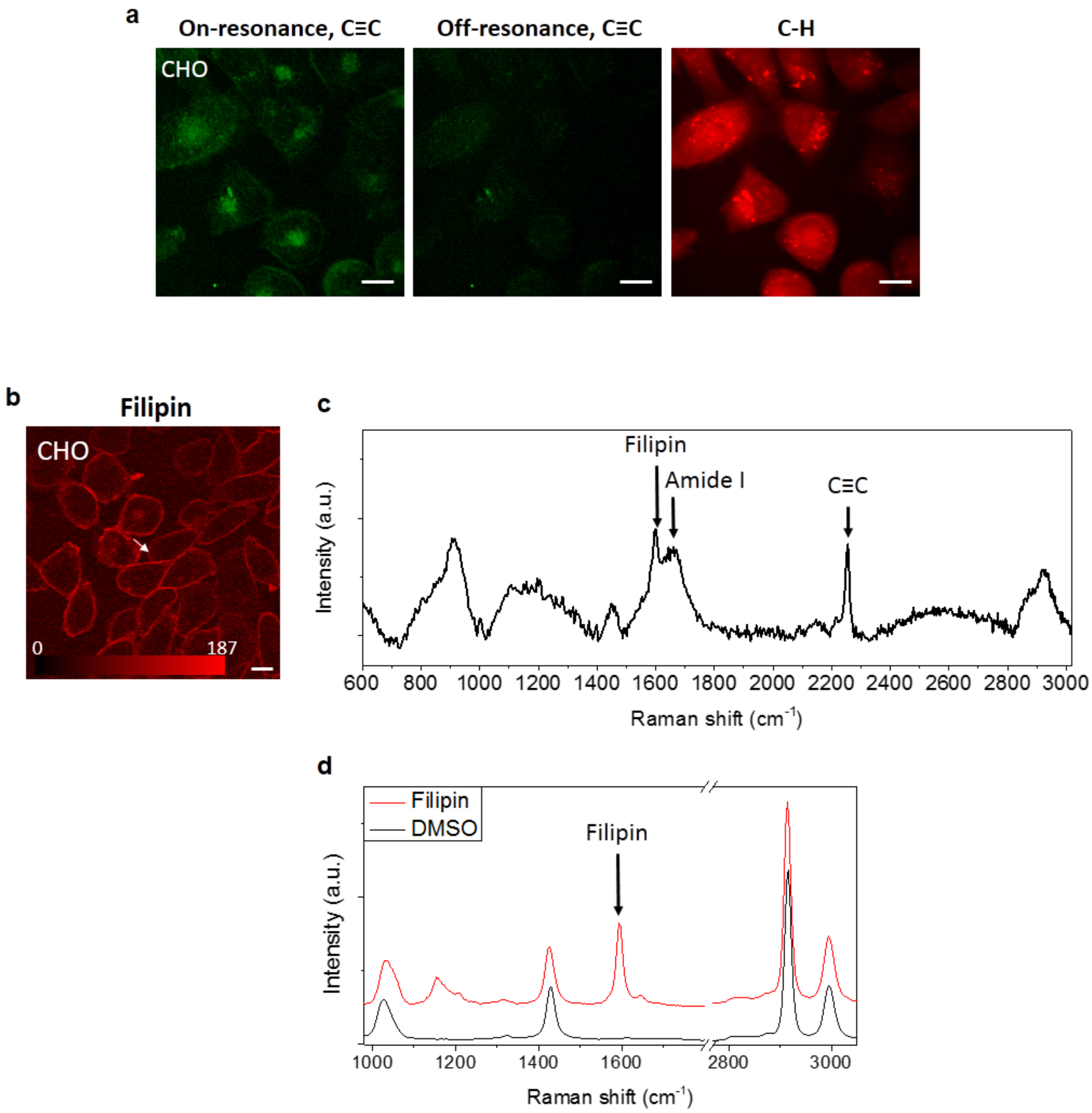

Supplementary Figure 4. PhDY-Chol is incorporated into cellular membrane. (a) SRS images of live CHO cells treated with PhDY-Chol for 1 h. PhDY-Chol was seen in plasma membrane and intracellular structures. Data acquisition time: 6 µs per pixel for 512 x 512 pixels. Scale bar: 10 µm. **(b)** TPEF image of filipin-labeled CHO cells. Arrow indicates the point used for Raman spectral analysis. Red intensity bar represents the relative intensity of fluorescence. Scale bar: 10 µm. **(c)** Raman spectrum of filipin-labeled cell membrane acquired on the same TPEF microscope. The bands for filipin, protein (amide I), and C≡C vibrational mode are indicated by black arrows. Spectrum acquisition time: 30 s. **(d)** Raman spectrum of filipin and the solvent, DMSO. The band for filipin is indicated by black arrows. Spectrum acquisition time: 10 s.

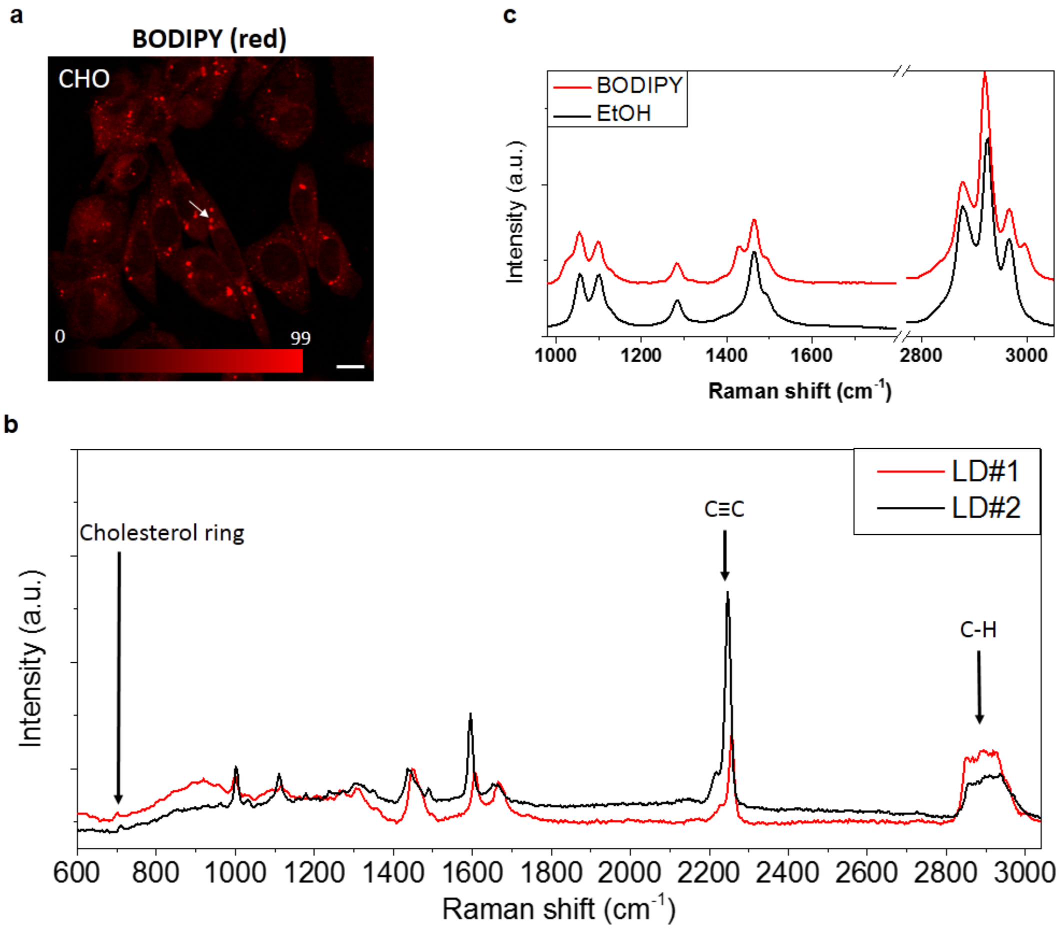

Supplementary Figure 5. TPEF imaging and Raman spectral analysis to confirm that PhDY-Chol is stored into LDs. (a) TPEF image of BODIPY-labeled CHO cells. Arrow indicates the point used for Raman spectral analysis. Red intensity bar represents the relative intensity of fluorescence. Scale bar: 10 μm. **(b)** Representative Raman spectra of BODIPY-labeled LDs acquired on the same TPEF microscope. The bands for cholesterol ring, C≡C, and C-H vibrational modes are indicated by black arrows. Two representative spectra of LDs are shown. Spectrum acquisition time: 30 s. **(c)** Raman spectrum of BODIPY and the solvent, ethanol. Spectrum acquisition time: 10 s.

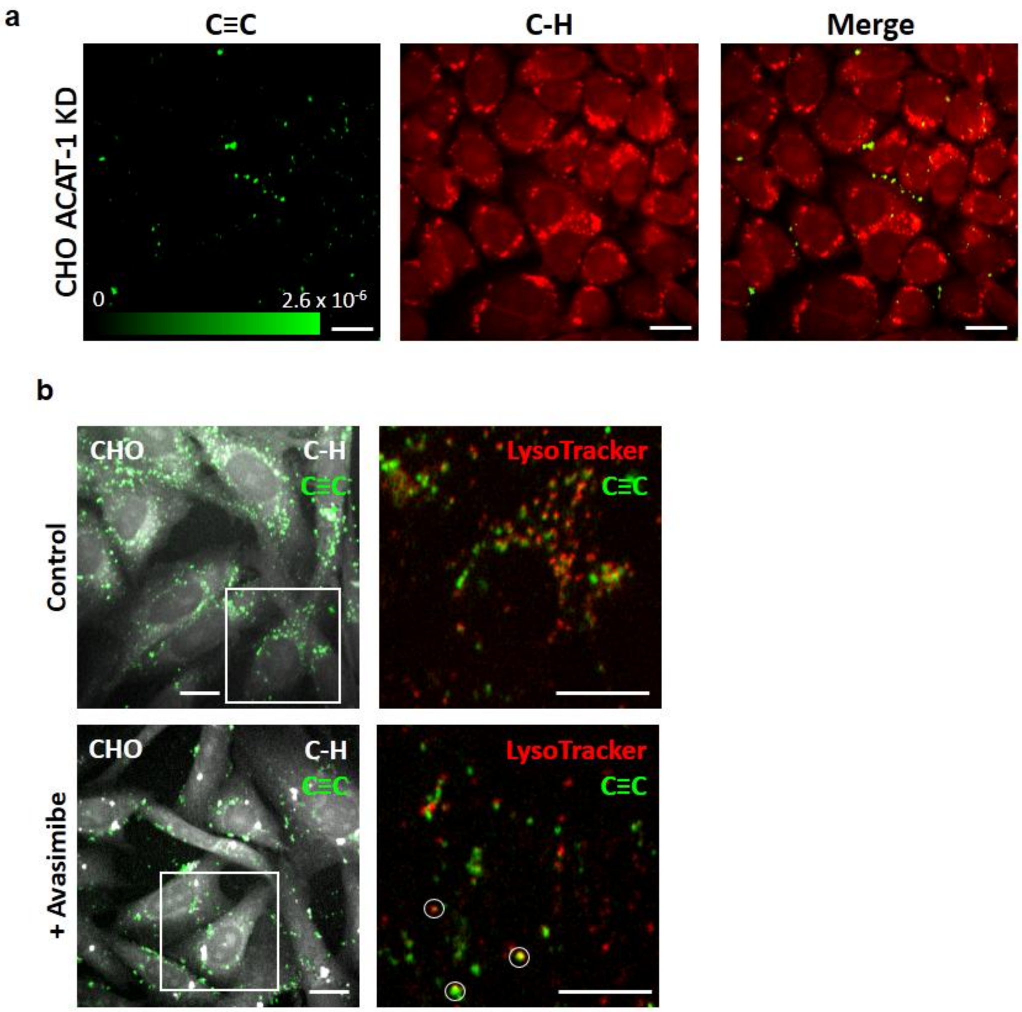

Supplementary Figure 6. ACAT-1 inhibition blocks PhDY-CHOL storage into LDs. (a) SRS images of ACAT-1 knocked down CHO cells. No overlap was observed between PhDY-rich particles and LDs. Intensity bar shows the ΔI/I value of the SRS image. Image acquisition speed: 10 µs per pixel for 400 x 400 pixels. Scale bar: 10 µm. **(b)** SRS images of PhDY-Chol and TPEF images of LysoTracker-stained organelles in CHO cells and ACAT-1 inhibited CHO cells by avasimibe treatment. PhDY-Chol was overlapped with LDs but not with LysoTracker-stained organelles in control CHO cells. After avasimibe treatment, PhDY-Chol was not overlapped with LDs but with LysoTracker-stained organelles (circles). Image acquisition speed: 10 µs per pixel for 400 x 400 pixels. Scale bar: 10 µm.

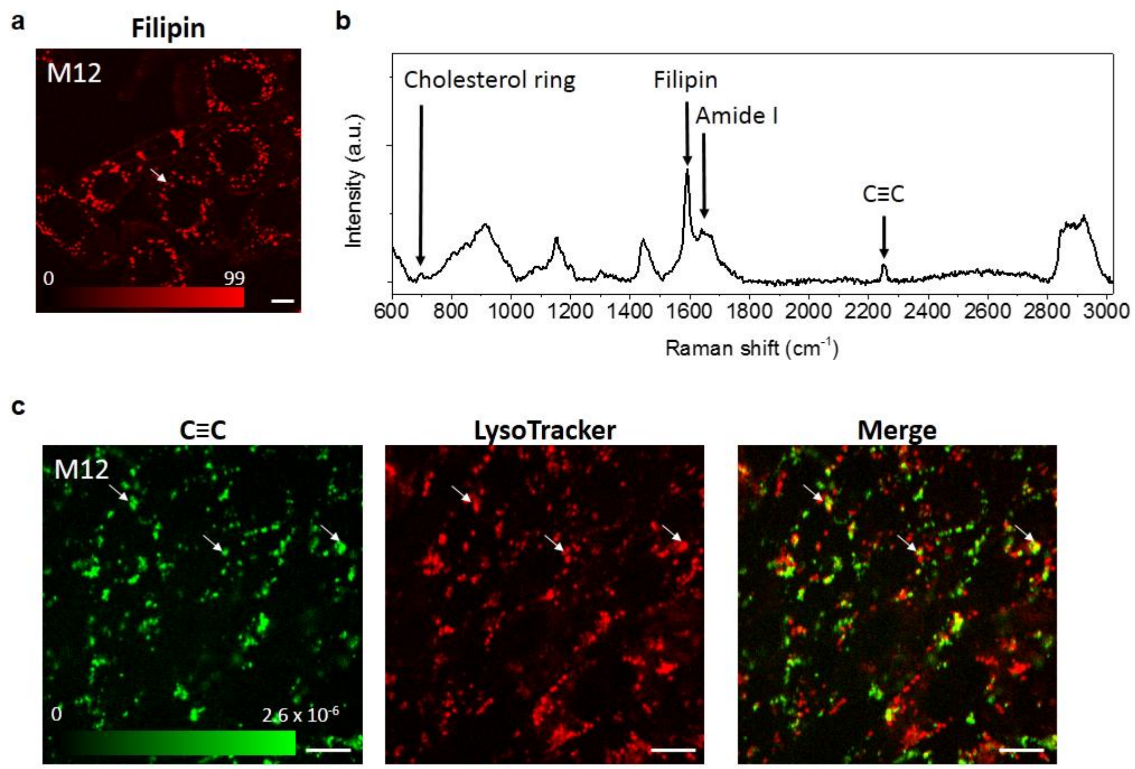

Supplementary Figure 7. PhDY-Chol reflects lysosomal cholesterol accumulation in M12 cells. (a) TPEF image of filipin-labeled M12 cells. Arrow indicates the point used for Raman spectral analysis. Red intensity bar represents the relative intensity of fluorescence. Image acquisition speed: 10 µs per pixel for 400 x 400 pixels. Scale bar: 10 µm. **(b)** Raman spectrum of the filipin-labeled organelle acquired on the same TPEF microscope. The bands for cholesterol ring, filipin, protein (amide I), and C≡C vibrational mode are indicated by black arrows. Spectrum acquisition time: 30 s. **(c)** SRS image of PhDY-Chol and TPEF image of LysoTracker-stained organelles in M12 cells. All PhDY-CHOL was found inside lysosomes. Arrows representatively indicate that PhDY-Chol is accumulated in lysosomes. Intensity bar shows the ΔI/I value of the image. Image acquisition speed: 10 µs per pixel for 400 x 400 pixels. Scale bar: 10 µm.

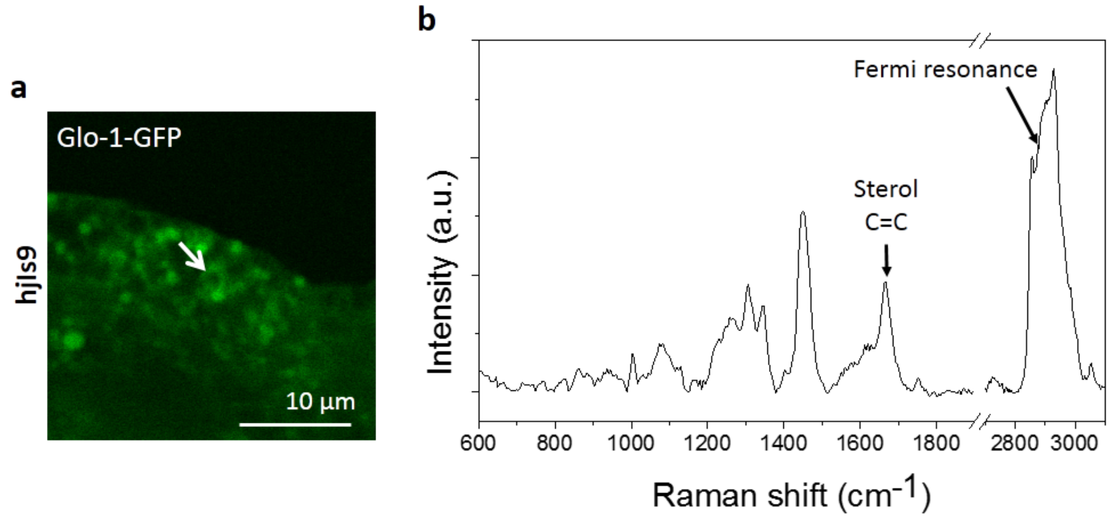

Supplementary Figure 8. TPEF imaging and Raman spectral analysis to confirm that cholesterol is stored in LDs. (a) TPEF image of hjIs9 worm, which contains GFP targeted to lysosome-related organelles (LROs) in intestinal cells. Arrow indicates the point used for Raman spectral analysis. **(b)** Raman spectrum of GFP surrounded LROs acquired on the same TPEF microscope. The bands for sterol C=C, and Fermi resonance between asymmetrical CH_2 vibrational modes are indicated by black arrow. Spectrum acquisition time: 10 s.

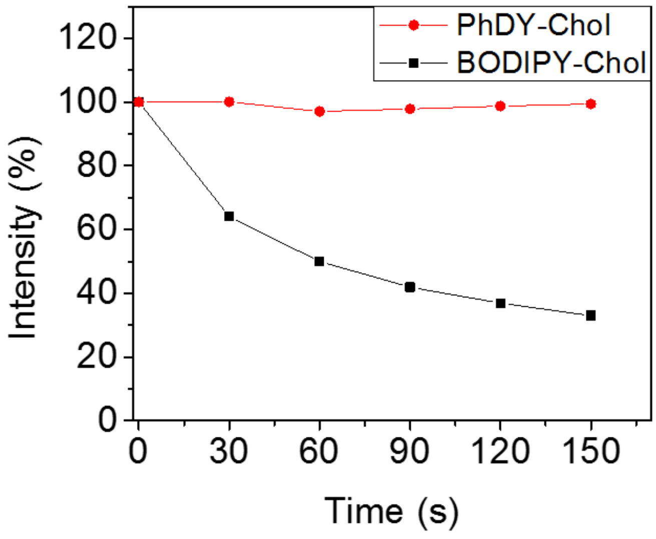

Supplementary Figure 9. The photostability of PhDY-Chol and the photo-bleaching of BODIPY-Chol. The SRS images of 50 mM PhDY-Chol solution and TPEF images of 50 mM BODIPY-Chol solution were acquired continuously for 150 s (one acquisition every 30 s). No significant change of the SRS signal was observed for PhDY-Chol, and a rapid photo-bleaching was observed for BODIPY-Chol.

Supplementary videos:

Supplementary Movie 1. SRS movie of real-time movement of PhDY-Chol stored in LDs in CHO cells. Image acquisition speed: 6.4 µs per pixel for 360 x 360 pixels. Scale bar: 10 µm.

Synthesis of Probes

General Methods. NMR spectra were recorded on (^{1}H at 400 MHz, 500 MHz and ^{13}C at 100 MHz, 125 MHz) spectrometers, Chemical shifts (*δ*) were given in ppm with reference to solvent signals [^{1}H NMR: $CDCl_3$ (7.26); ^{13}C NMR: $CDCl_3$ (77.2)]. Column chromatography was performed on silica gel. All reactions sensitive to air or moisture were carried out under argon atmosphere in dry and freshly distilled solvents under anhydrous conditions, unless otherwise noted. Anhydrous THF was distilled over sodium benzophenone ketyl under N_2. Anhydrous CH_2Cl_2 was distilled over calcium hydride under N_2. Anhydrous MeOH was distilled over magnesium under N_2. All other solvents and reagents were used as obtained from commercial sources without further purification.

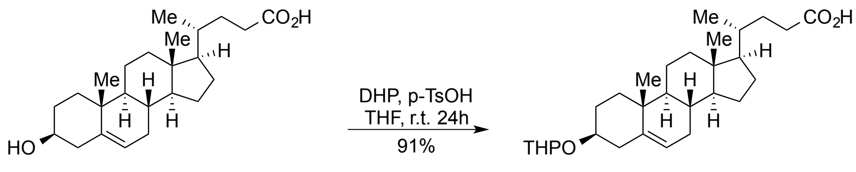

Compound S1. To a solution of acid **3** (200 mg, 0.53 mmol) in dry THF (22.5 mL) were added 3,4-dihydro-2H-pyran (DHP) (0.24 mL, 2.65 mmol) and p-toluenesulfonic acid monohydrate (p-TsOH) (20.2 mg, 0.11 mmol) under argon. After the mixture was stirred at room temperature for 24 h, saturated aqueous $NaHCO_3$ solution and CH_2Cl_2 were added to quench the reaction. The aqueous layer was extracted with CH_2Cl_2 (3 × 50 mL), and the combined organic layers were acidified with acetic acid (15 mL), washed with water (3 × 50 mL) and dried with Na_2SO_4. The solvent was removed under vacuum, and the residue was purified by chromatography (Hexane/EtOAc, 8:1) to give **S1** (222 mg, 91%) as a white solid.

^{1}H NMR (500 MHz, $CDCl_3$): *δ* 5.35-5.33 (m, 1H), 4.74-4.71 (m, 1H), 3.93-3.90 (m, 1H), 3.54-3.46 (m, 1H), 2.42-2.19 (m, 4H), 1.98-1.71 (m, 9H), 1.61-1.43 (m, 12H), 1.35-1.07 (m, 7H), 1.00 (s, 3H), 0.93 (d, *J* = 7.0 Hz, 3H), 0.67 (s, 3H); ^{13}C NMR (100 MHz, $CDCl_3$): *δ* 180.0, 141.2, 121.7, 97.1, 96.9, 76.2, 63.0, 62.9, 56.9, 55.9, 50.3, 42.5, 40.4, 39.9, 38.9, 37.6, 37.4, 36.9, 35.5, 32.0, 31.4, 31.1, 30.9, 29.8, 28.2, 28.2, 28.1, 25.6, 24.4, 21.2, 20.1, 19.5, 18.4, 12.0; IR (film): 2938, 1708, 1454, 1200, 1059, 1033, 975 cm^{-1}; MS (ESI): *m/z* 457.4 [M-H]$^-$.

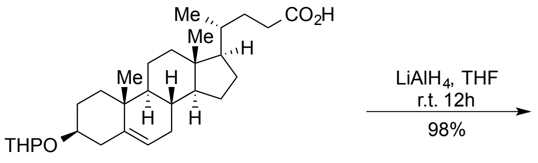

S1 **S2**

Compound S2. A solution of **S1** (222 mg, 0.48 mmol) in dry THF (12 mL) was added dropwise to a suspension of $LiAlH_4$ (55 mg, 1.45 mmol) in dry THF (8 mL) under argon at 0 °C. After the addition, the reaction mixture was allowed to warm to room temperature and stirred overnight. Saturated aqueous $NaHCO_3$ solution (10 mL) was added slowly to quench the reaction, and the resulting mixture was extracted with CH_2Cl_2 (3 × 60 mL). The combined organic layers were washed with brine and dried with Na_2SO_4. The solvent was removed under vacuum, and the residue was purified by chromatography (Hexane/EtOAc, 4:1) to give **S2** (209 mg, 98%) as a white solid. ^{1}H NMR (400 MHz, $CDCl_3$): δ 5.32 (t, J = 6.0 Hz, 1H), 4.70 (s, 1H), 3.93-3.86 (m, 1H), 3.58 (td, J = 9.2, 2.8 Hz, 2H), 3.52-3.45 (m, 2H), 2.34-2.18 (m, 2H), 1.97-1.81 (m, 8H), 1.57-1.38 (m, 16H), 1.23-1.05 (m, 6H), 0.98 (s, 3H), 0.92 (d, J = 6.4 Hz, 3H), 0.66 (s, 3H); ^{13}C NMR (100 MHz, $CDCl_3$): δ 141.2, 141.0, 121.6, 121.6, 97.1, 96.9, 76.1, 63.6, 63.0, 62.9, 56.8, 56.1, 50.3, 50.2, 42.4, 40.3, 39.9, 38.9, 37.6, 37.3, 36.9, 36.9, 35.7, 32.0, 32.0, 31.4, 29.8, 29.5, 28.3, 28.1, 25.6, 24.4, 21.2, 20.2, 20.1, 19.5, 18.8, 12.0; IR (film): 2936, 1456, 1377, 1112, 1059, 1033 cm^{-1}; MS (ESI): *m/z* 467.4 $[M+Na]^+$.

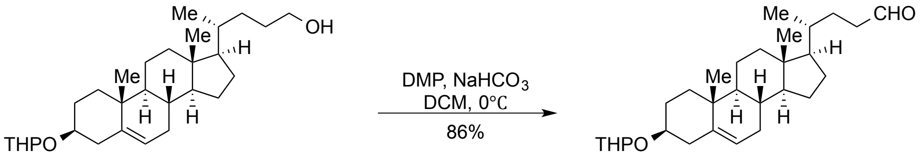

S2 **S3**

Compound S3. To a suspension of **S2** (79 mg, 0.18 mmol) and $NaHCO_3$ (45 mg, 0.53 mmol) in CH_2Cl_2 (4 mL) was added freshly prepared Dess-Martin Periodinane (113 mg, 0.27 mmol) at 0 °C. The reaction mixture was stirred at 0 °C. After the reaction is complete, saturated aqueous $NaHCO_3$ solution (10 mL) and saturated aqueous $Na_2S_2O_3$ solution (10 mL) were added and allowed to stir for 30 min at room temperature. Two layers were separated and the aqueous layer was washed with

EtOAc (2 × 40 mL). The combined organic layers were washed with saturated aqueous $NaHCO_3$ solution (2 × 30 mL), brine and dried with Na_2SO_4. The solvent was removed under vacuum, and the residue was purified by chromatography (Hexane/EtOAc, 4:1) to give **S3** (68 mg, 86%) as a white solid.

^{1}H NMR (400 MHz, $CDCl_3$): δ 9.75 (t, J = 2.0 Hz, 1H), 5.33 (td, J = 5.2, 1.6 Hz, 1H), 4.70 (t, J = 3.6 Hz, 1H), 3.93-3.87 (m, 1H), 3.55-3.44 (m, 2H), 2.44-2.18 (m, 4H), 1.99-1.93 (m, 2H), 1.84-1.69 (m, 6H), 1.57-1.42 (m, 12H), 1.33-1.05 (m, 7H), 0.99 (s, 3H), 0.91 (d, J = 6.4 Hz, 3H), 0.66 (s, 3H); ^{13}C NMR (100 MHz, $CDCl_3$): δ 203.2, 141.2, 142.0, 121.6, 121.5, 97.1, 96.9, 76.1, 63.0, 63.0, 56.8, 55.9, 50.2, 50.2, 42.5, 41.0, 40.4, 39.8, 38.9, 37.6, 37.3, 36.9, 36.9, 35.4, 32.0, 31.4, 29.8, 28.3, 28.1, 25.6, 24.4, 21.1, 20.2, 20.1, 19.5, 18.5, 12.9; IR (film): 2936, 2867, 1726, 1440, 1199, 1134, 1033, 1025, 976 cm^{-1}; MS (ESI): *m/z* 441.1 $[M-H]^-$.

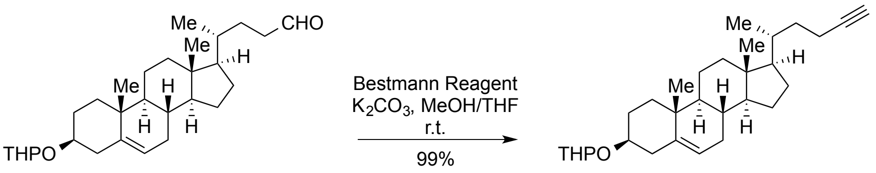

S3 4

Compound 4. To a solsution of **S3** (415 mg, 0.914 mmol) and K_2CO_3 (504.6 mg, 3.66 mmol) in dry methanol (14 mL) and THF (14 mL) were added dimethyl-1-diazo-2-oxopropylphosphonate (Bestmann Reagent, 0.33 mL, 2.194 mmol) and stirred under room temperature. After the reaction was complete, the reaction was diluted with EtOAc, washed with saturated aqueous $NaHCO_3$ solution, and dried over $MgSO_4$. The solvent was removed under vacuum, and the residue was purified by chromatography (Hexane/EtOAc, 20:1) to give **4** (397 mg, 99%) as a white solid.

^{1}H NMR (500 MHz, $CDCl_3$): δ 5.35-5.32 (m, 1H), 4.71-4.70 (m, 1H), 3.93-3.88 (m, 1H), 3.53-3.46 (m, 2H), 2.35-2.09 (m, 4H), 2.00-1.82 (m, 7H), 1.73-1.70 (m, 2H), 1.64-1.40 (m, 12H), 1.30-1.05 (m, 7H), 1.00 (s, 3H), 0.92 (d, J = 6.5 Hz, 3H), 0.68 (s, 3H); ^{13}C NMR (125 MHz, $CDCl_3$): δ 141.2, 141.0, 121.6, 121.6, 97.1, 96.9, 85.3, 76.1, 68.0, 63.0, 62.9, 56.8, 56.0, 50.3, 50.2, 42.6, 40.4, 39.9, 38.9, 37.6, 37.3, 36.9, 36.9, 35.3, 35.0, 32.0, 32.0, 31.4, 28.3, 28.1, 25.6, 24.4, 21.2, 20.2, 20.2, 19.5, 18.3, 15.6, 12.0; IR (film): 3311, 2935, 2868, 2850, 2118, 1466, 1454, 1440, 1376, 1114, 1057, 869 cm^{-1}; MS (ESI): *m/z* 461.4 $[M+Na]^+$.

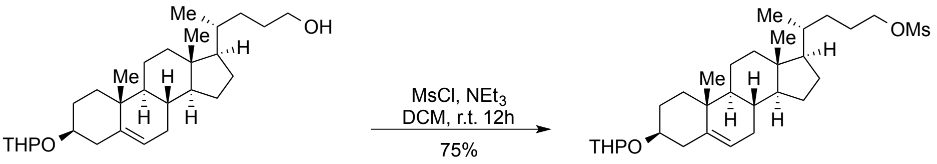

S2 **S4**

Compound S4. To a solution of **S2** (100 mg, 0.225 mmol) and triethylamine (0.094 mL, 0.676 mmol) in CH_2Cl_2 (5 mL) was added methanesulfonyl chloride (77.4 mg, 0.676 mmol) under 0 °C. After the addition, the reaction mixture was allowed to warm to room temperature and stirred overnight. Saturated aqueous $NaHCO_3$ solution was added to quench the reaction, and the resulting mixture was extracted with ethyl ether. The combined organic layers were washed with brine and dried with $MgSO_4$. The solvent was removed under vacuum, and the residue was purified by chromatography (Hexane/EtOAc, 8:1) to give **7** (88 mg, 75%) as a white solid.

^{1}H NMR (400 MHz, $CDCl_3$): δ 5.33 (t, J = 4.8 Hz, 1H), 4.70 (s, 1H), 4.19 (dd, J =10.0, 5.2 Hz, 2H), 3.92-3.89 (m, 1H), 3.54-3.46 (m, 2H), 2.99 (s, 3H), 2.35-2.16 (m, 2H), 2.00-1.94 (m, 2H), 1.85-1.79 (m, 5H), 1.70-1.44 (m, 15H), 1.24-1.08 (m, 7H), 1.00 (s, 3H), 0.93 (d, J = 5.2 Hz, 3H), 0.67 (s, 3H); ^{13}C NMR (125 MHz, $CDCl_3$): δ 141.2, 141.0, 121.6, 121.6, 97.1, 97.0, 76.1, 70.8, 63.0, 63.0, 56.8, 55.9, 50.2, 42.5, 40.4, 39.9, 38.9, 37.5, 37.3, 36.9, 35.4, 32.0, 31.6, 31.4, 29.8, 28.3, 28.1, 26.0, 25.6, 25.6, 24.4, 21.2, 20.2, 19.5, 18.7, 12.0; IR (film): 2937, 2881, 2858, 1355, 1034, 962, 837 cm^{-1}; MS (ESI): *m/z* 545.4 [M+Na]$^+$.

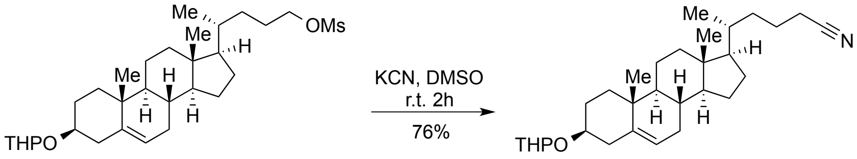

S4 **S5**

Compound S5. To a solution of **S4** (40 mg, 0.077 mmol) in DMSO (1.5 mL) was added potassium cyanide (10 mg, 0.153 mmol). After the addition, the reaction mixture was heated to 90 °C and stirred for 2 h. Water (5 mL) was added to quench the reaction, and the resulting mixture was extracted with ethyl acetate (4 × 4 mL). The combined organic layers were washed with brine and dried with $MgSO_4$. The solvent was removed under vacuum, and the residue was purified by chromatography (Hexane/EtOAc, 8:1) to give **S4** (27 mg, 76%) as a white solid.

^{1}H NMR (500 MHz, $CDCl_3$): δ 5.34 (dd, J = 6.8, 4.4 Hz, 1H), 4.72-4.70 (m, 1H), 3.94-3.89 (m, 1H), 3.55-3.46 (m, 2H), 2.36-2.16 (m, 4H), 2.00-1.94 (m, 2H), 1.87-1.83 (m, 4H), 1.73-1.68 (m, 2H), 1.62-1.41 (m, 14H), 1.30-1.04 (m, 7H), 1.00 (s, 3H), 0.93 (d, J = 6.5 Hz, 3H), 0.68 (s, 3H); ^{13}C NMR (125 MHz, $CDCl_3$): δ 141.2, 141.0, 121.6, 121.6, 120.0, 97.1, 97.0, 76.1, 63.1, 63.0, 56.8, 56.0, 50.3, 50.2, 42.5, 40.4, 39.9, 39.0, 37.6, 37.3, 36.9, 36.9, 35.4, 35.2, 32.0, 31.4, 29.8, 28.3, 28.1, 25.6, 24.4, 22.3, 21.2, 20.2, 20.2, 19.5, 18.7, 17.7, 12.0; IR (film): 2924, 2854, 2352, 2323, 1456, 1033, 1021, 973 cm^{-1}; MS (ESI): *m/z* 476.4 $[M+Na]^{+}$.

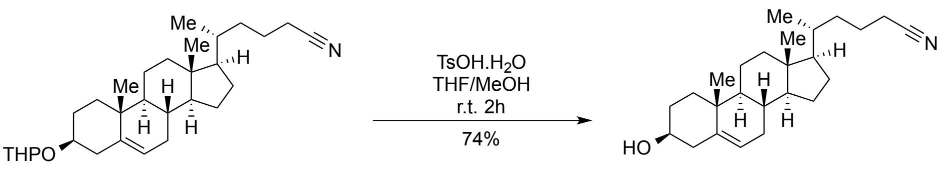

S5 **8**

Compound 8. **S5** (20 mg, 0.044 mmol) and p-toluenesulfonic acid monohydrate (1.68 mg, 0.009 mmol) were dissolved in THF (0.5 mL) and methanol (0.5 mL), and stirred under room temperature. After the reaction is complete (2 h), the reaction mixture was diluted with ethyl ether, washed with saturated aqueous $NaHCO_3$ solution, and dried with $MgSO_4$. The solvent was removed under vacuum, and the residue was purified by chromatography (Hexane/EtOAc, 4:1) to give **8** (16 mg, 74%) as a white solid.

^{1}H NMR (400 MHz, $CDCl_3$): δ 5.35 (dt, J =5.2, 2.0 Hz, 1H), 3.53 (tt, J = 11.2, 4.4 Hz, 1H), 2.33-2.23 (m, 4H), 2.01-1.95 (m, 2H), 1.86-1.69 (m, 5H), 1.63-1.41 (m, 10 H), 1.29-1.05 (m, 7H), 1.00 (s, 3H), 0.94 (d, J = 6.4 Hz, 3H), 0.68 (s, 3H); ^{13}C NMR (100 MHz, $CDCl_3$): δ 140.9, 121.8, 120.0, 71.9, 56.9, 55.9, 50.2, 42.5, 42.4, 39.9, 37.4, 36.6, 35.4, 35.2, 32.0, 31.8, 28.4, 24.4, 22.4, 21.2, 19.5, 18.7, 17.7, 12.0; IR (film): 2939, 2890, 2345, 2316, 1465, 1063, 960 cm^{-1}; MS (ESI): *m/z* 368.2 $[M-H]^{-}$.

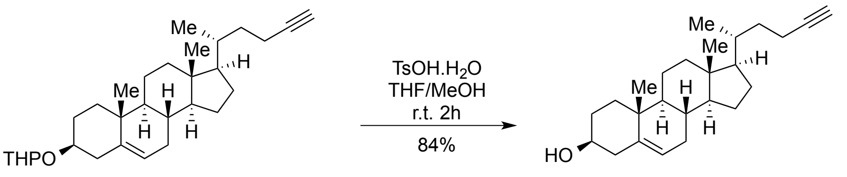

4 **5**

Compound 5. Compound **4** (53 mg, 0.121 mmol) and p-toluenesulfonic acid monohydrate (4.6 mg, 0.024 mmol) were dissolved in THF (1 mL) and methanol (1 mL), and stirred under room temperature. After the reaction is complete (2 h), the reaction mixture was diluted with ethyl ether, washed with saturated aqueous $NaHCO_3$ solution, and dried with $MgSO_4$. The solvent was removed under vacuum, and the residue was purified by chromatography (Hexane/EtOAc, 10:1 to 4:1) to give **5** (36 mg, 84%) as a white solid.

^{1}H NMR (400 MHz, $CDCl_3$): δ 5.34 (dd, J = 4.4, 2.0 Hz, 1H), 3.51 (tt, J = 11.6, 4.0 Hz, 1H), 2.31-2.05 (m, 4H), 2.01-1.91 (m, 3H), 1.87-1.80 (m, 3H), 1.73-1.66 (m, 2H), 1.60-1.40 (m, 8H), 1.33-1.07 (m, 7H), 1.00 (s, 3H), 0.92 (d, J = 6.8 Hz, 3H), 0.68 (s, 3H); ^{13}C NMR (100 MHz, $CDCl_3$): δ 140.9, 121.8, 85.3, 71.9, 68.0, 56.9, 56.0, 50.2, 42.6, 42.4, 39.9, 37.4, 36.6, 35.3, 34.9, 32.0, 31.8, 28.3, 24.4, 21.2, 19.5, 18.3, 15.6, 12.0; IR (film): 3302, 2935, 2852, 2334, 1465, 1377, 1135, 1051, 801 cm^{-1}; MS (ESI): *m/z* 353.2 $[M-H]^{-}$.

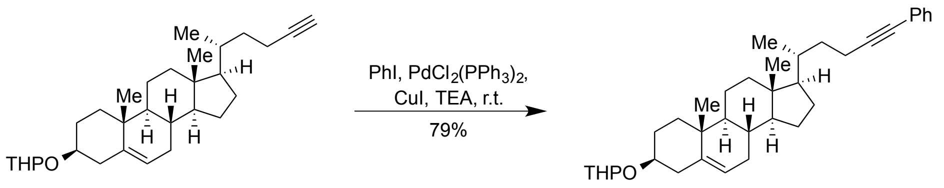

4 **S6**

Compound S6. The mixture of $PdCl_2(PPh_3)_2$ (2.2 mg, 0.003 mmol), CuI (0.6 mg, 0.003 mmol), and iodobenzene (7.2 μL, 0.064 mmol) in triethylamine (0.2 mL) was bubbled with Argon gas for ten minutes. To the mixture, triethylamine solution (0.2 mL) of **4** (27.6 mg, 0.063 mmol) was added at room temperature. After stirring for 5 h at room temperature, the reaction was quenched with saturated NH_4Cl aqueous solution and extracted with ethyl acetate. The organic layer was washed with brine, dried over $MgSO_4$. The solvent was removed under vacuum, and the residue was purified by chromatography (Hexane/EtOAc, 40:1) to give **S6** (26 mg, 79%) as a white solid.

^{1}H NMR (400 MHz, $CDCl_3$): δ 7.38 (d, J = 5.2 Hz, 2H), 7.27-7.26 (m, 3H), 5.35 (m, 1H), 4.72 (s, 1H), 3.92 (s, 1H), 3.50 (t, J = 14.4 Hz, 2H), 2.49-2.17 (m, 4H), 2.03-1.71 (m, 9H), 1.56-1.44 (m, 9H), 1.34-1.04 (m, 9H), 1.01 (s, 3H), 0.98 (d, J = 6.8 Hz, 3H), 0.7 (s, 3H); ^{13}C NMR (100 MHz, $CDCl_3$): δ 141.2, 141.1, 131.6, 128.3, 127.6, 124.3,

121.7, 97.1, 97.0, 91.0, 80.5, 76.1, 63.1, 63.0, 56.9, 56.0, 50.3, 42.6, 40.4, 39.9, 38.9, 37.6, 37.4, 36.9, 35.5, 35.2, 32.1, 31.4, 29.8, 28.4, 28.1, 25.6, 24.4, 21.2, 20.3, 20.2, 19.5, 18.5, 16.7, 12.0; IR (film): 2942, 2864, 2333, 1492, 1201, 1136, 1024, 972cm^{-1}; MS (ESI): *m/z* 537.4 [M+Na]$^{+}$.

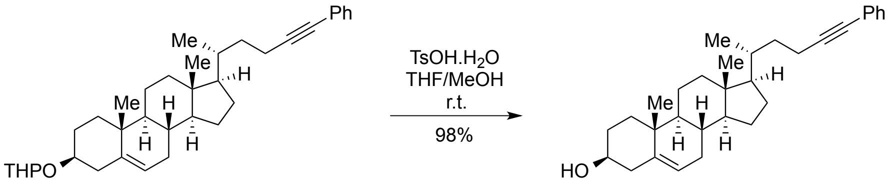

Compound 6. **S6** (26 mg, 0.05 mmol) and p-toluenesulfonic acid monohydrate (9.5 mg, 0.05 mmol) were dissolved in THF (1 mL) and methanol (1 mL), and stirred under room temperature. After the reaction was complete, the reaction mixture was diluted with ethyl ether, washed with saturated aqueous $NaHCO_3$ solution, and dried with $MgSO_4$. The solvent was removed under vacuum, and the residue was purified by chromatography (Hexane/EtOAc, 4:1) to give **6** (21 mg, 98%) as a white solid.

^{1}H NMR (400 MHz, $CDCl_3$): δ 7.38 (d, J = 5.6 Hz, 2H), 7.27-7.26 (m, 3H), 5.35 (s, 1H), 3.55-3.50 (m, 1H), 2.49-2.20 (m, 4H), 2.04-1.74 (m, 7H), 1.58-1.45 (m, 8H), 1.34-1.08 (m, 7H), 1.01 (s, 3H), 0.98 (d, J = 6.4 Hz, 3H), 0.70 (s, 3H); ^{13}C NMR (100 MHz, $CDCl_3$): δ 140.9, 131.6, 128.3, 127.6, 124.3, 121.8, 91.0, 80.5, 71.9, 56.9, 56.0, 50.2, 42.6, 42.4, 39.9, 37.4, 36.6, 35.5, 35.2, 32.0, 31.8, 28.3, 24.4, 21.2, 19.5, 18.5, 16.7, 12.0; IR (film): 2934, 2851, 2325, 1597, 1466, 1376, 1132, 1108, 1040cm^{-1}; MS (ESI): *m/z* 453.2 [M+Na]$^{+}$.

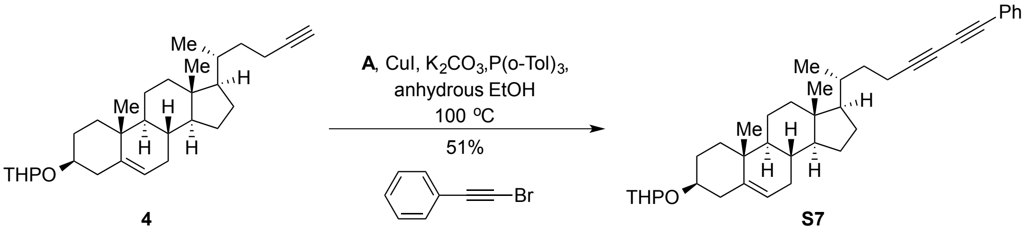

Compound S7. A vial was charged with CuI (1.7 mg, 0.009 mmol), P(o-Tol)$_3$ (5.5 mg, 0.018 mmol), K_2CO_3 (24.9 mg, 0.18 mmol), **4** (40 mg, 0.09 mmol), compound **A** (freshly synthesized according to the literature route,[3] 21.2 mg, 0.117 mmol), and

anhydrous EtOH (3 mL). The mixture was stirred for 12 h at 100 °C then cooled to room temperature. The mixture was diluted with EtOAc, filtered through a pad of Celite, and concentrated under vacuo. The residue was purified by chromatography (Hexane/EtOAc, 20:1) to give **S7** (25 mg, 51%) as a white solid.

^{1}H NMR (400 MHz, $CDCl_3$): δ 7.47 (dd, J = 4.0, 2.0 Hz, 2H), 7.35-7.27 (m, 3H), 5.35 (t, J = 6.0 Hz, 1H), 4.72 (t, J = 4.0 Hz, 1H), 3.56-3.45 (m, 2H), 2.37-2.23 (m, 4H), 2.02-1.94 (m, 2H), 1.86-1.83 (m, 5H), 1.75-1.71 (m, 2H), 1.59-1.44 (m, 10 H), 1.32-1.08 (m, 8H), 1.01 (s, 3H), 0.94 (d, J = 6.8 Hz, 3H), 0.70 (s, 3H); ^{13}C NMR (100 MHz, $CDCl_3$): δ 141.2, 141.0, 132.6, 128.9, 128.4, 122.3, 121.6, 121.6, 97.1, 97.0, 85.4, 76.2, 74.8, 74.6, 65.0, 63.0, 63.0, 56.9, 56.0, 50.3, 50.3, 42.6, 40.4, 39.9, 38.9, 37.6, 37.4, 36.9, 36.9, 35.4, 34.7, 32.0, 31.4, 29.8, 28.3, 28.1, 25.6, 24.4, 21.2, 20.2, 20.2, 19.5, 18.3, 16.8, 12.0; IR (film): 2938, 2868, 2325, 1508, 1456, 1116, 1026, 755 cm^{-1}; MS (ESI): *m/z* 537.3 [M-H]$^-$.

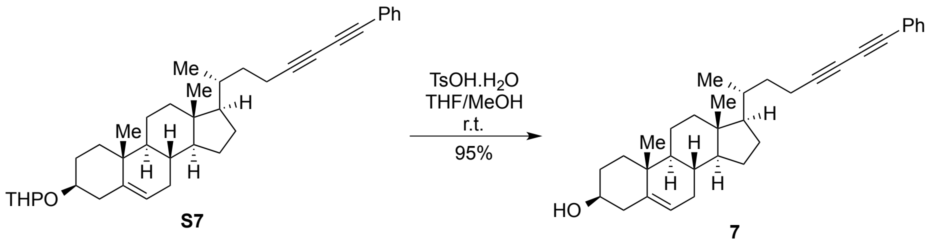

S7 **7**

Compound 7. **S7** (25 mg, 0.046 mmol) and p-toluenesulfonic acid monohydrate (1.70 mg, 0.010 mmol) were dissolved in THF (1 mL) and methanol (1 mL), and stirred under room temperature. After the reaction was complete, the reaction mixture was diluted with ethyl ether, washed with saturated aqueous $NaHCO_3$ solution, and dried with $MgSO_4$. The solvent was removed under vacuum, and the residue was purified by chromatography (Hexane/EtOAc, 4:1) to give **7** (20 mg, 95%) as a white solid.

^{1}H NMR (400 MHz, $CDCl_3$): δ 7.47 (dd, J = 4.0, 2.0 Hz, 2H), 7.33-7.29 (m, 3H), 5.36-5.34 (m, 1H), 3.52 (tt, J = 11.2, 4.8 Hz, 1H), 2.44-2.20 (m, 4H), 2.02-1.95 (m, 2H), 1.85-1.71 (m, 4H), 1.59-1.41 (m, 8H), 1.32-1.08 (m, 8H), 1.01 (s, 3H), 0.94 (d, J = 6.4 Hz, 3H), 0.70 (s, 3H); ^{13}C NMR (100 MHz, $CDCl_3$): δ 141.0, 132.6, 128.9, 128.5, 122.3, 121.8, 85.4, 74.8, 74.6, 71.9, 65.0, 56.9, 56.0, 50.2, 42.6, 42.4, 39.9, 37.4, 36.6, 35.5, 34.7, 32.0, 31.8, 28.3, 24.4, 21.2, 19.5, 18.3, 16.8, 12.0; IR (film): 2925, 2853, 2343, 1465, 1377, 1108, 1059 cm^{-1}; MS (ESI): *m/z* 478 [M+Na]$^+$.

Supplementary References

1 Gordon, M. S. & Schmidt, M. W. in *Theory and Applications of Computational Chemistry* (eds Clifford E. Dykstra, Gernot Frenking, Kwang S. Kim, & Gustavo E. Scuseria) 1167-1189 (Elsevier, 2005).

2 Shao, Y., Molnar, L. F., Jung, Y., Kussmann, J., Ochsenfeld, C., Brown, S. T., Gilbert, A. T., Slipchenko, L. V., Levchenko, S. V., O'Neill, D. P., DiStasio, R. A., Jr., Lochan, R. C., Wang, T., Beran, G. J., Besley, N. A., Herbert, J. M., Lin, C. Y., Van Voorhis, T., Chien, S. H., Sodt, A., Steele, R. P., Rassolov, V. A., Maslen, P. E., Korambath, P. P., Adamson, R. D., Austin, B., Baker, J., Byrd, E. F., Dachsel, H., Doerksen, R. J., Dreuw, A., Dunietz, B. D., Dutoi, A. D., Furlani, T. R., Gwaltney, S. R., Heyden, A., Hirata, S., Hsu, C. P., Kedziora, G., Khalliulin, R. Z., Klunzinger, P., Lee, A. M., Lee, M. S., Liang, W., Lotan, I., Nair, N., Peters, B., Proynov, E. I., Pieniazek, P. A., Rhee, Y. M., Ritchie, J., Rosta, E., Sherrill, C. D., Simmonett, A. C., Subotnik, J. E., Woodcock, H. L., 3rd, Zhang, W., Bell, A. T., Chakraborty, A. K., Chipman, D. M., Keil, F. J., Warshel, A., Hehre, W. J., Schaefer, H. F., 3rd, Kong, J., Krylov, A. I., Gill, P. M. & Head-Gordon, M. Advances in methods and algorithms in a modern quantum chemistry program package. *Phys. Chem. Chem. Phys.* **8**, 3172-3191, (2006).

NMR Spectra of New Compounds

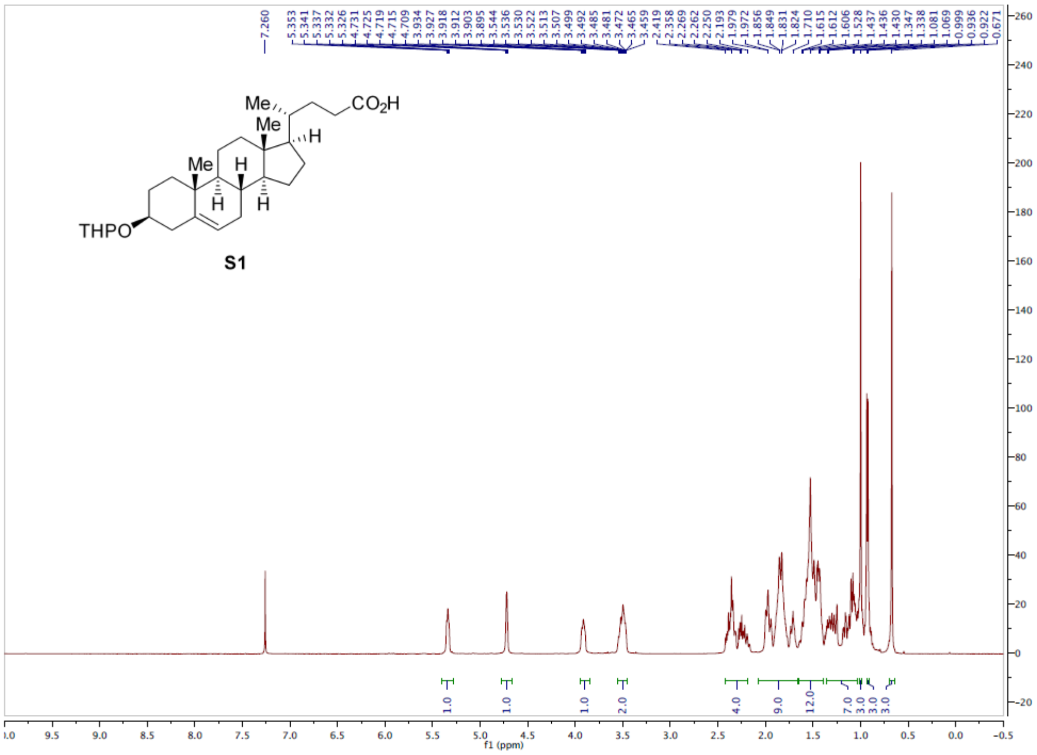

Supplementary Figure 9. ^{1}H NMR of **S1** (500 MHz, $CDCl_3$)

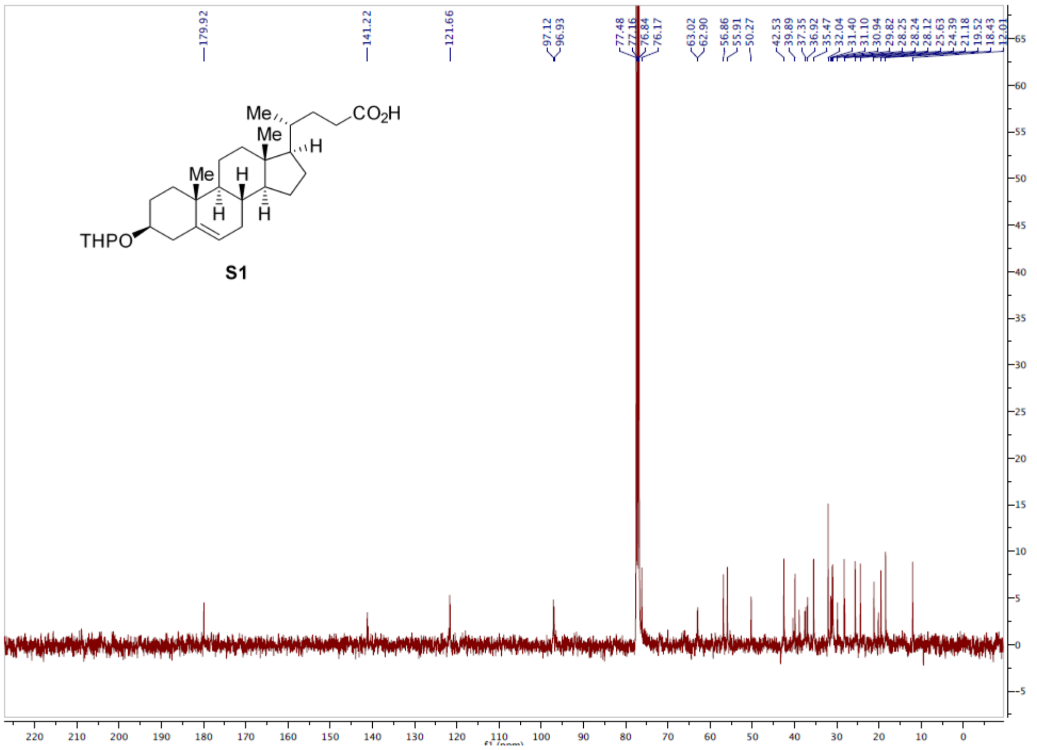

Supplementary Figure 10. ^{13}C NMR of **S1** (100 MHz, $CDCl_3$)

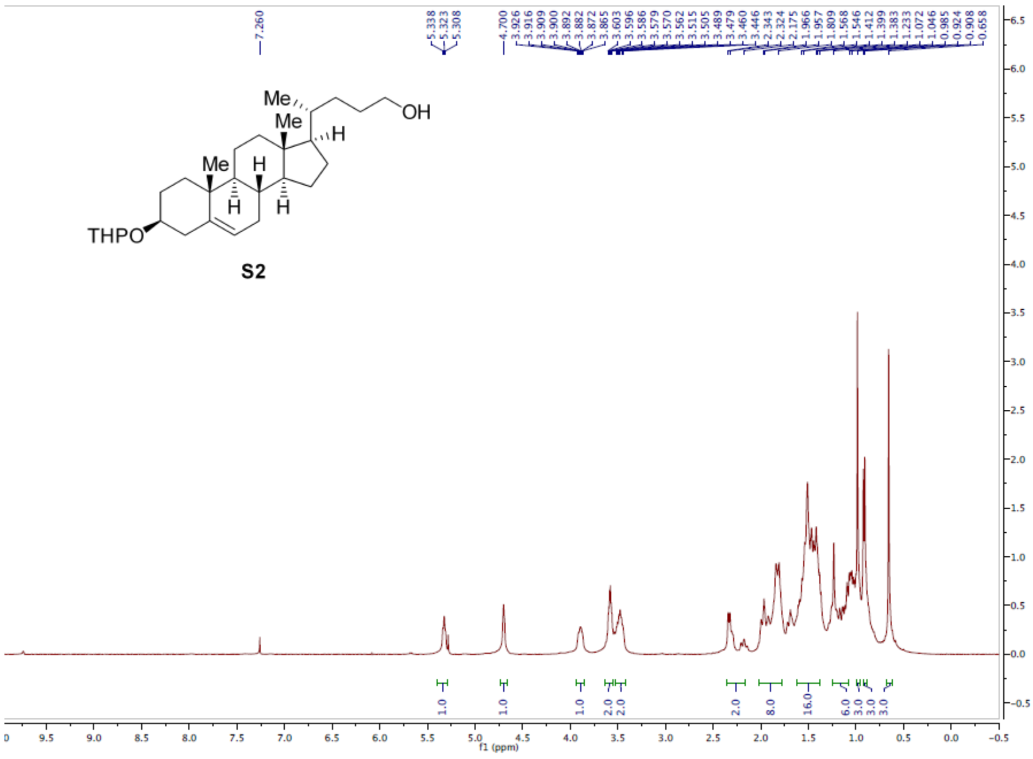

Supplementary Figure 11. ^{1}H NMR of **S2** (400 MHz, $CDCl_3$)

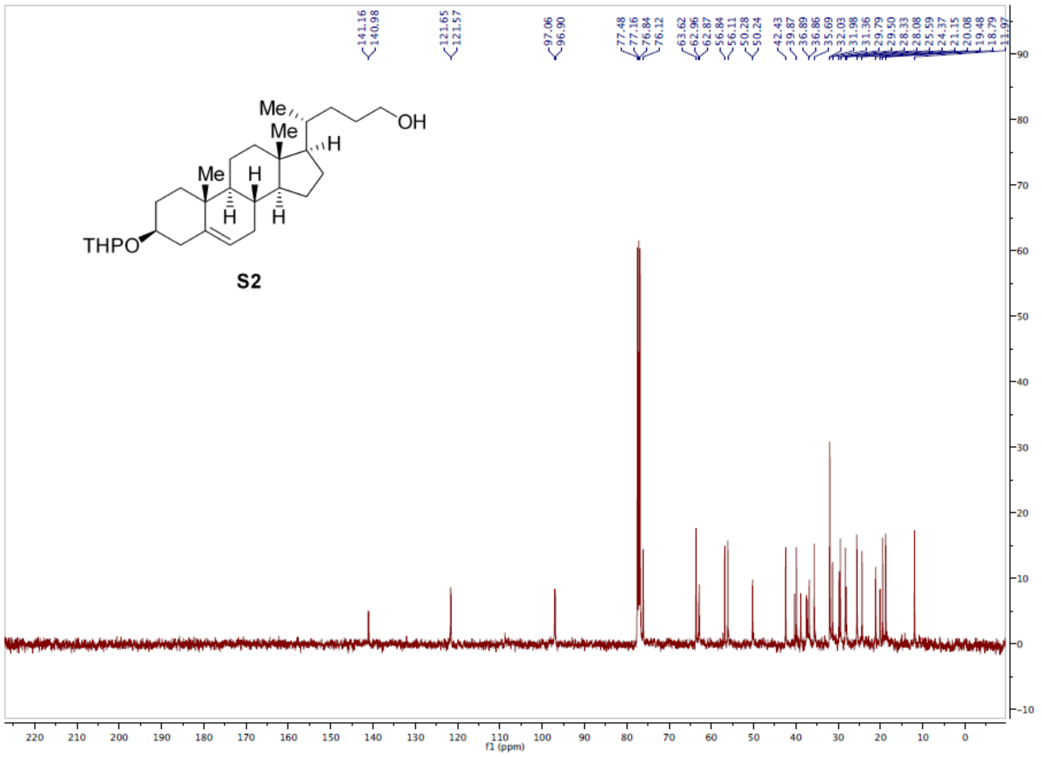

Supplementary Figure 12. ^{13}C NMR of **S2** (100 MHz, $CDCl_3$)

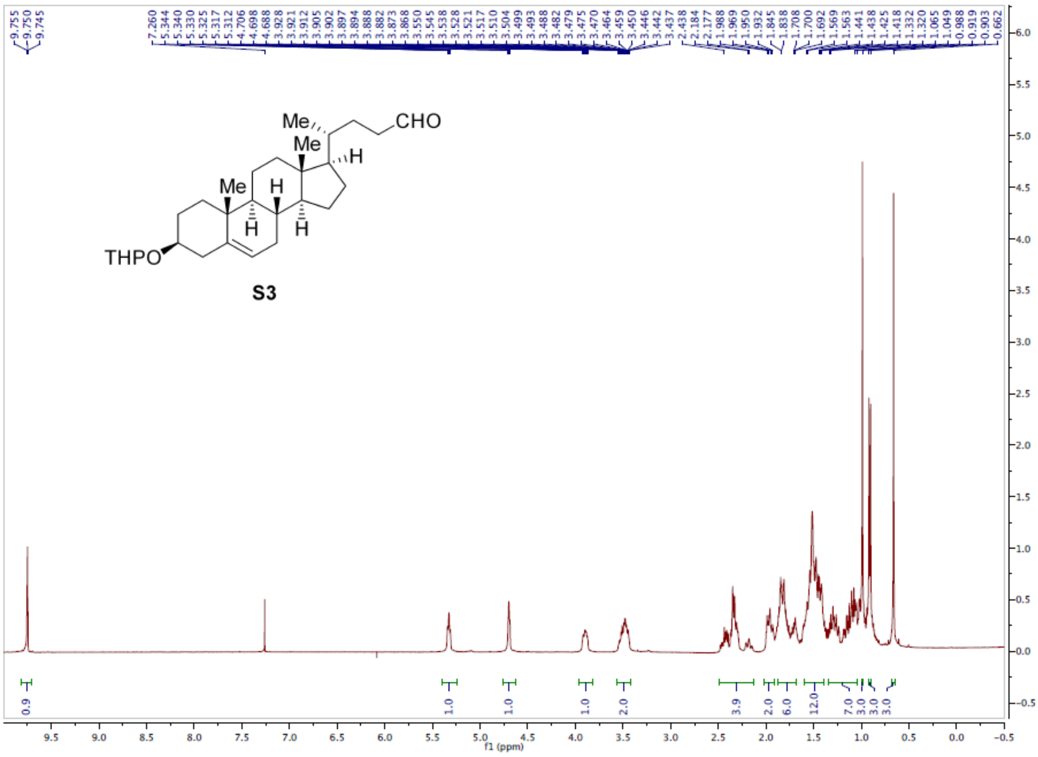

Supplementary Figure 13. ^{1}H NMR of **S3** (400 MHz, $CDCl_3$)

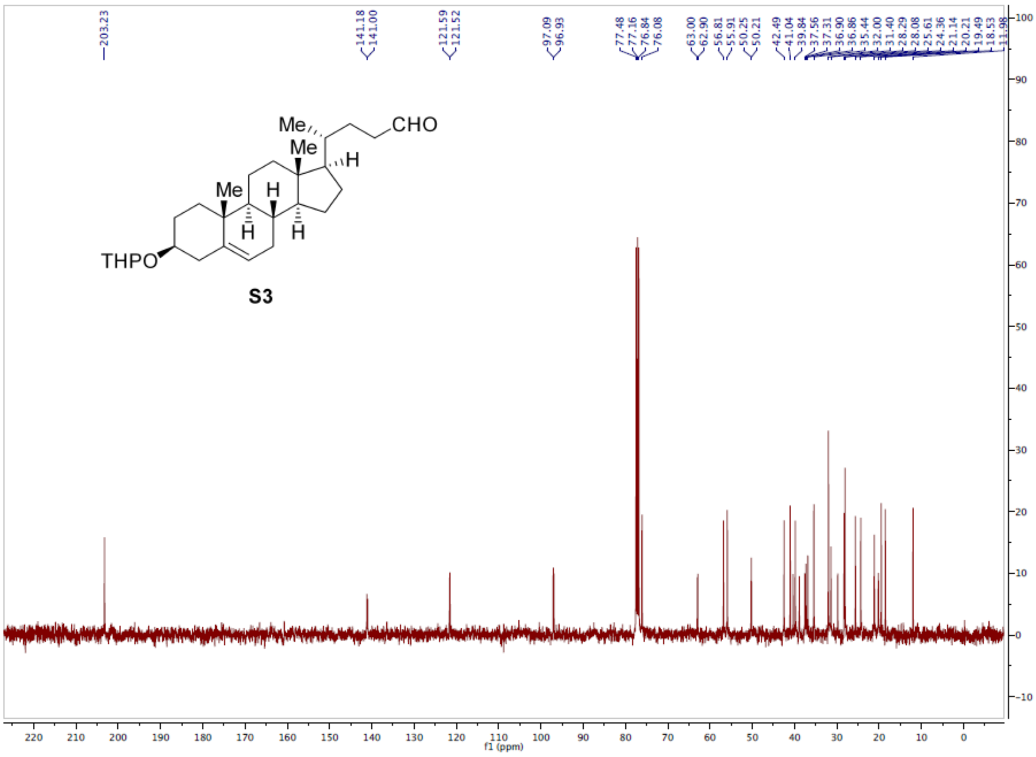

Supplementary Figure 14. ^{13}C NMR of **S3** (100 MHz, $CDCl_3$)

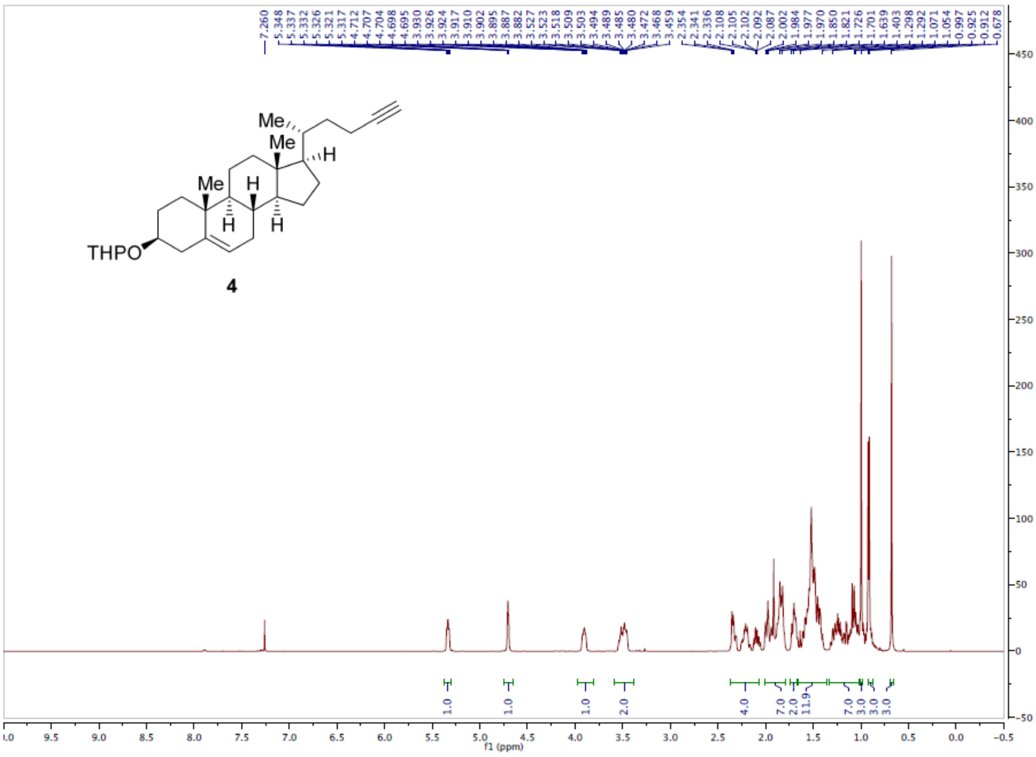

Supplementary Figure 15. ^{1}H NMR of **4** (500 MHz, $CDCl_3$)

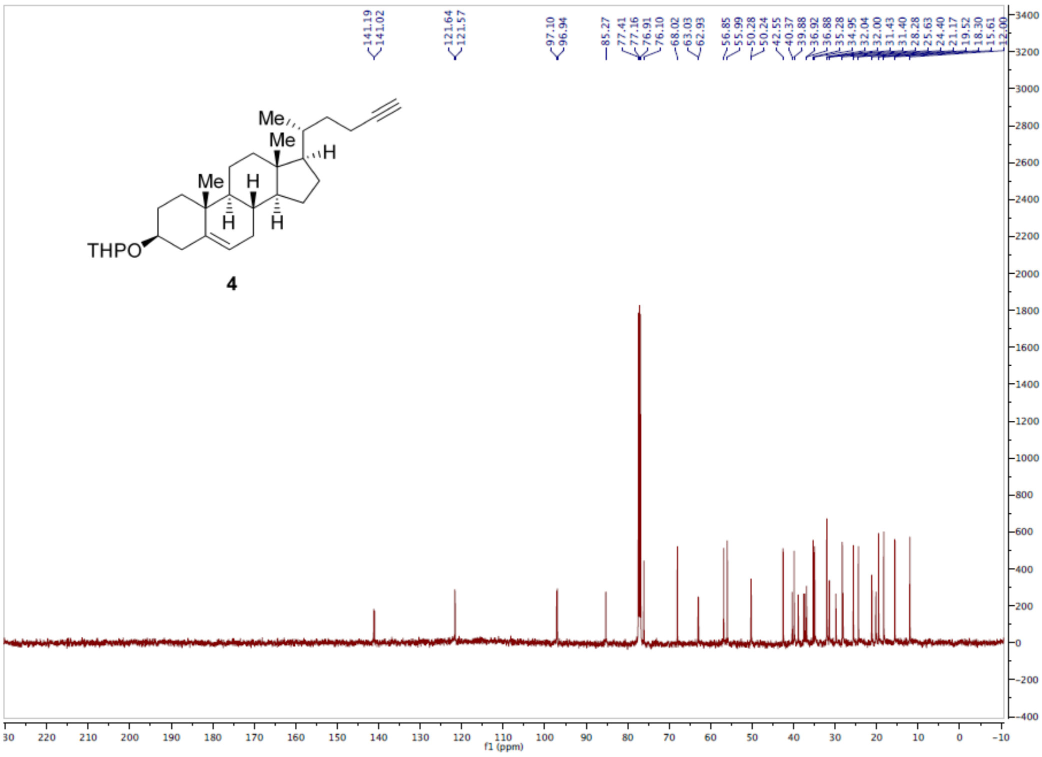

Supplementary Figure 16. ^{13}C NMR of **4** (125 MHz, $CDCl_3$)

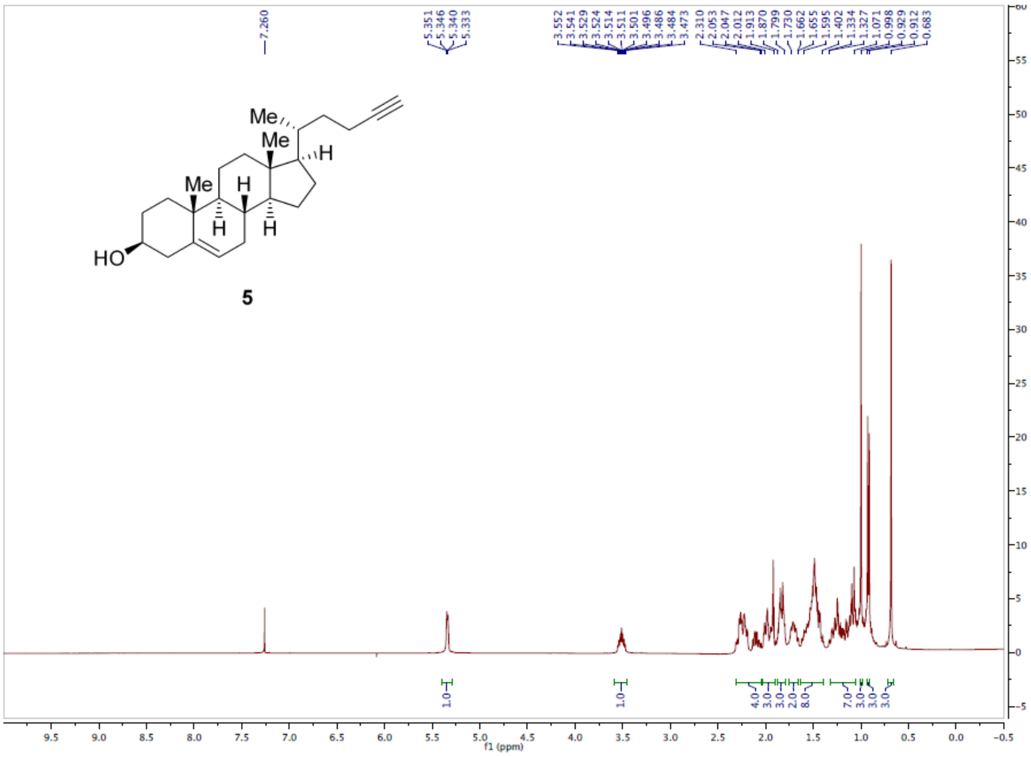

Supplementary Figure 17. ^{1}H NMR of **5** (400 MHz, $CDCl_3$)

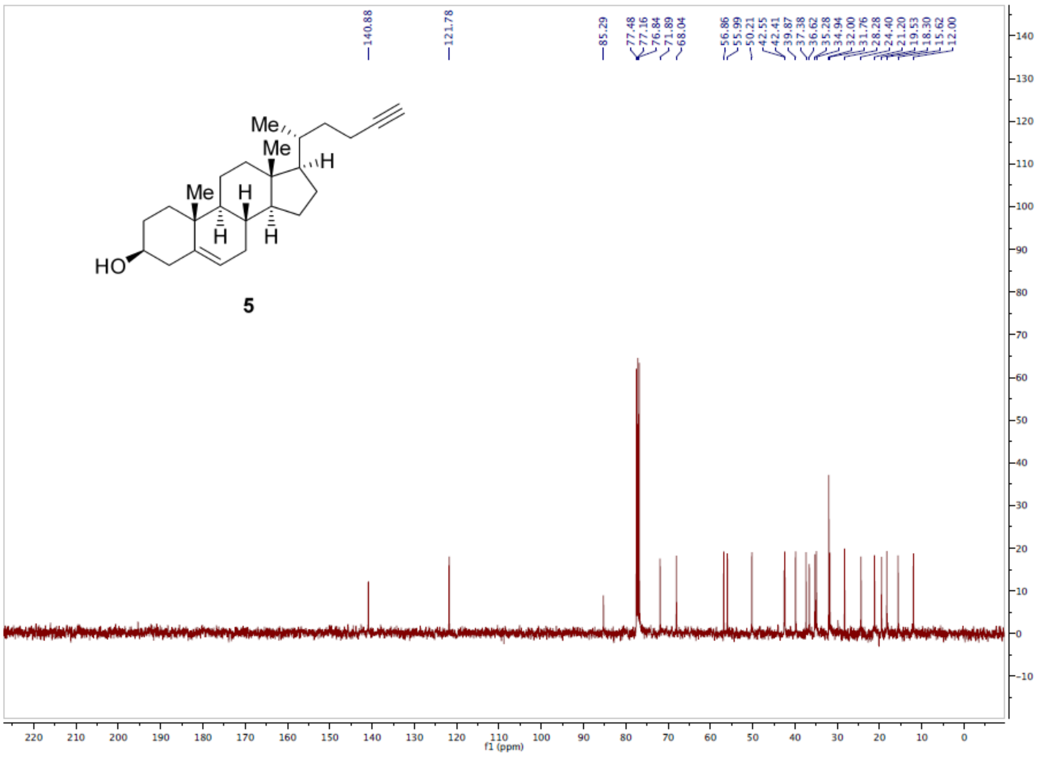

Supplementary Figure 18. ^{13}C NMR of **5** (100 MHz, $CDCl_3$)

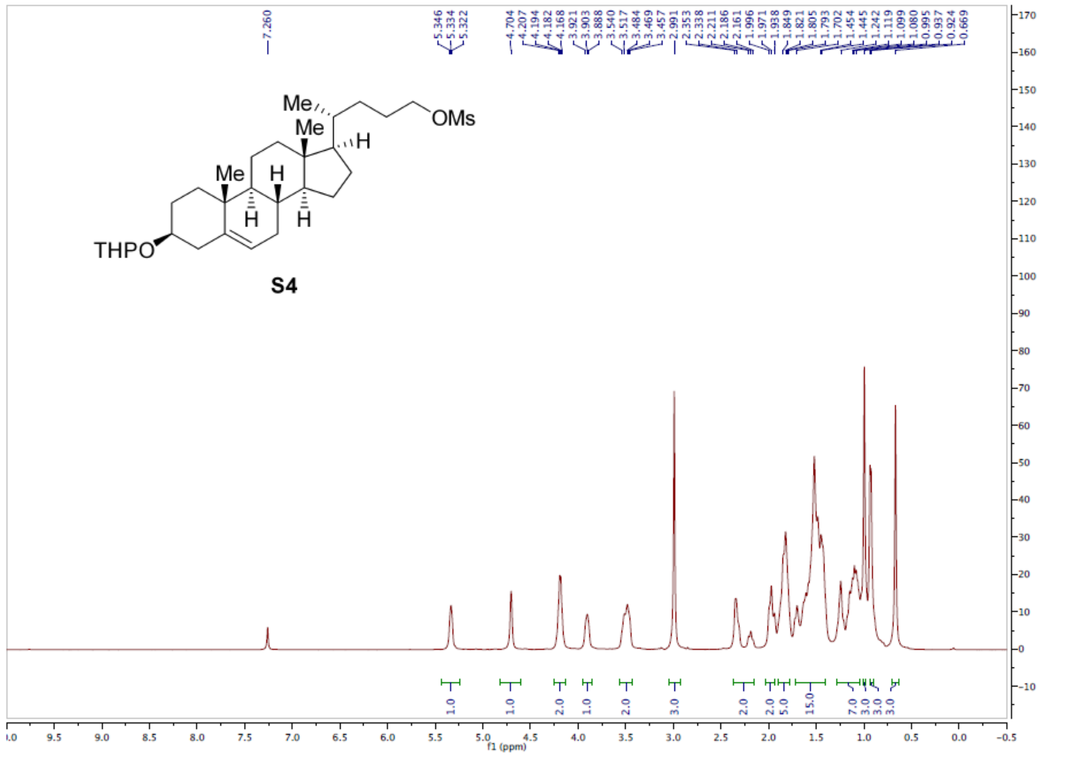

Supplementary Figure 19. ^{1}H NMR of **S4** (400 MHz, $CDCl_3$)

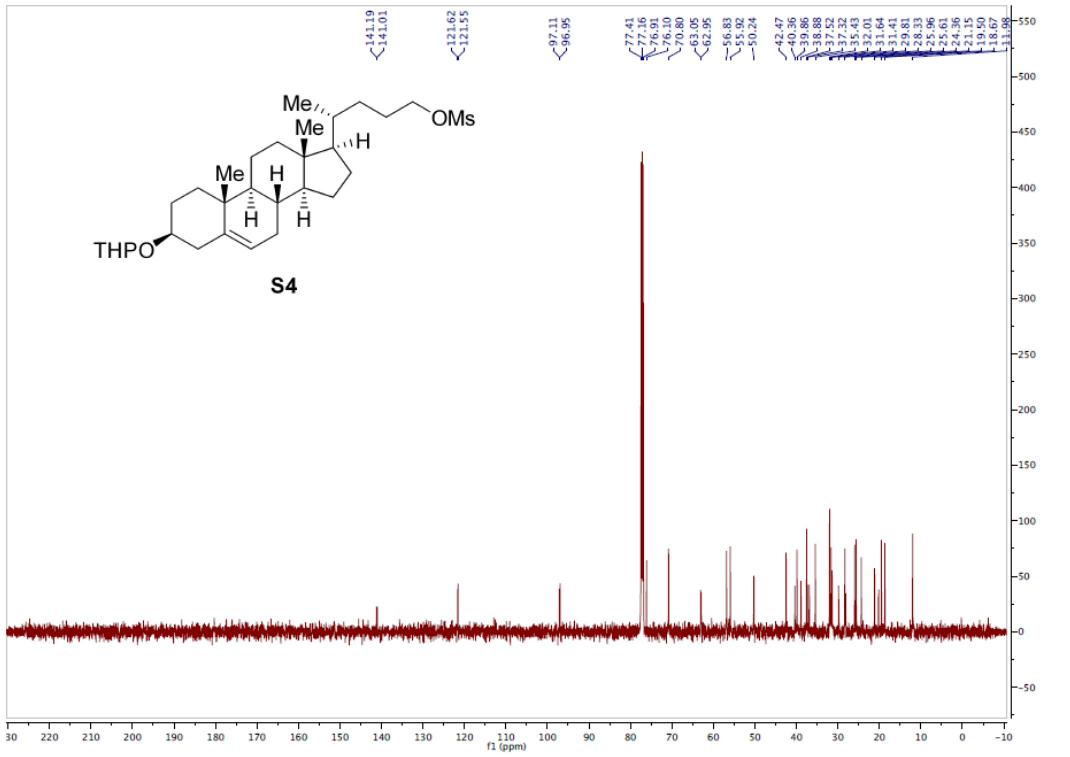

Supplementary Figure 20. ^{13}C NMR of **S4** (125 MHz, $CDCl_3$)

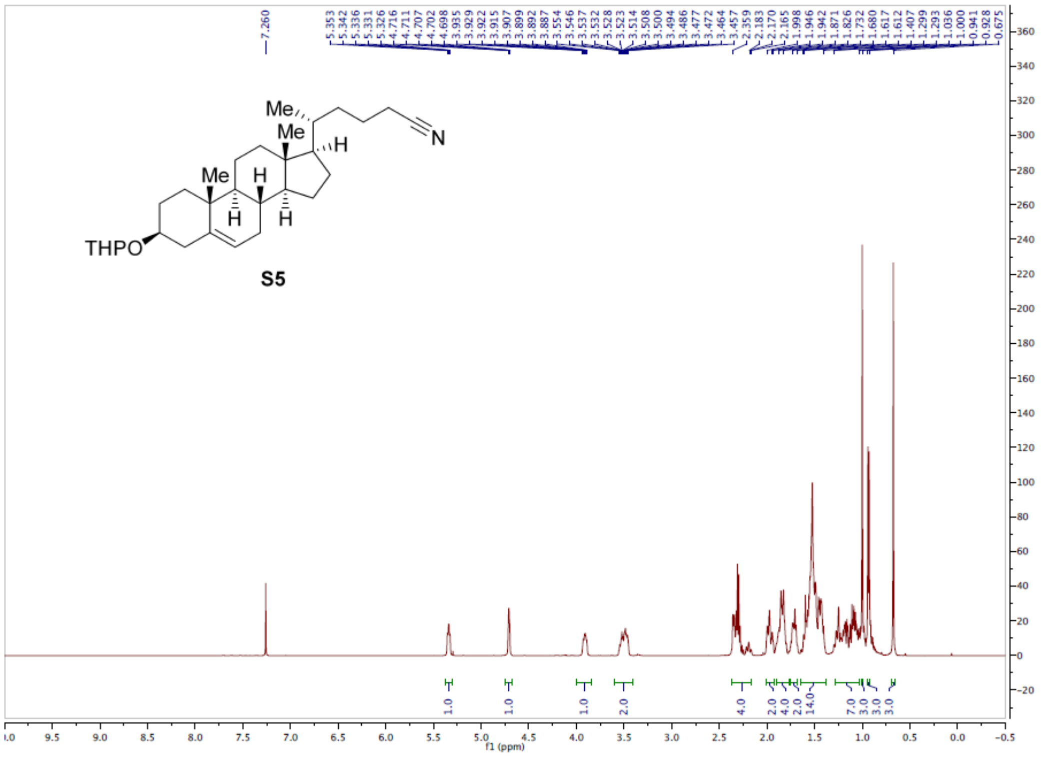

Supplementary Figure 21. ^{1}H NMR of **S5** (500 MHz, $CDCl_3$)

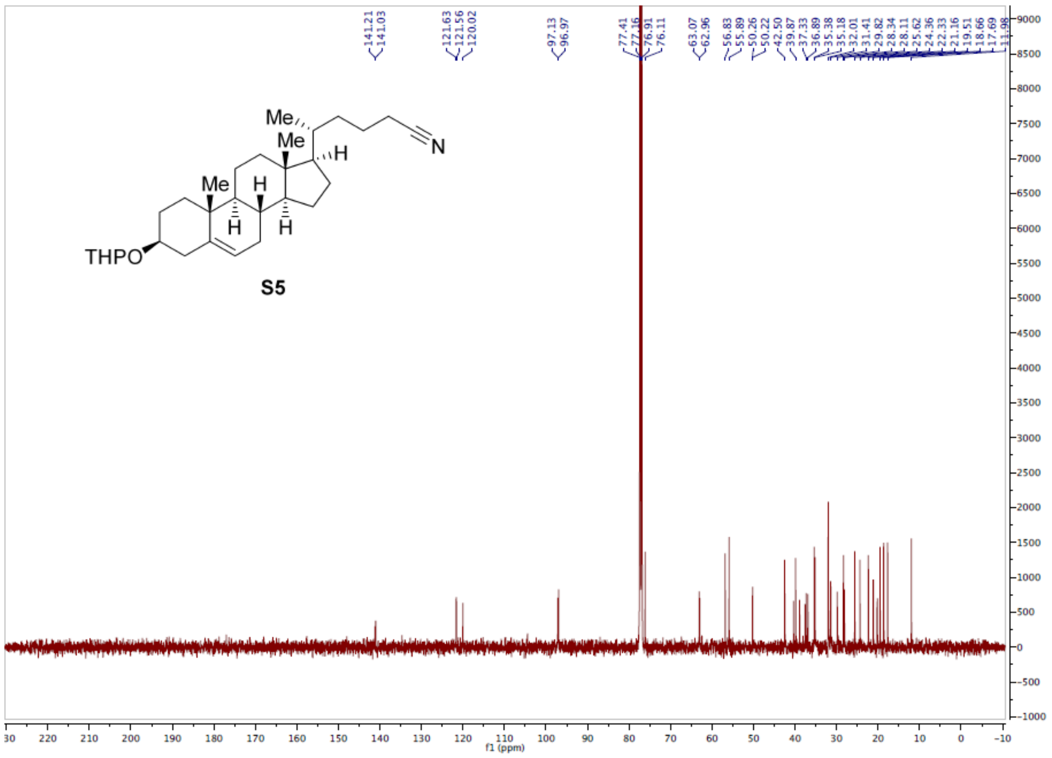

Supplementary Figure 22. ^{13}C NMR of **S5** (125 MHz, $CDCl_3$)

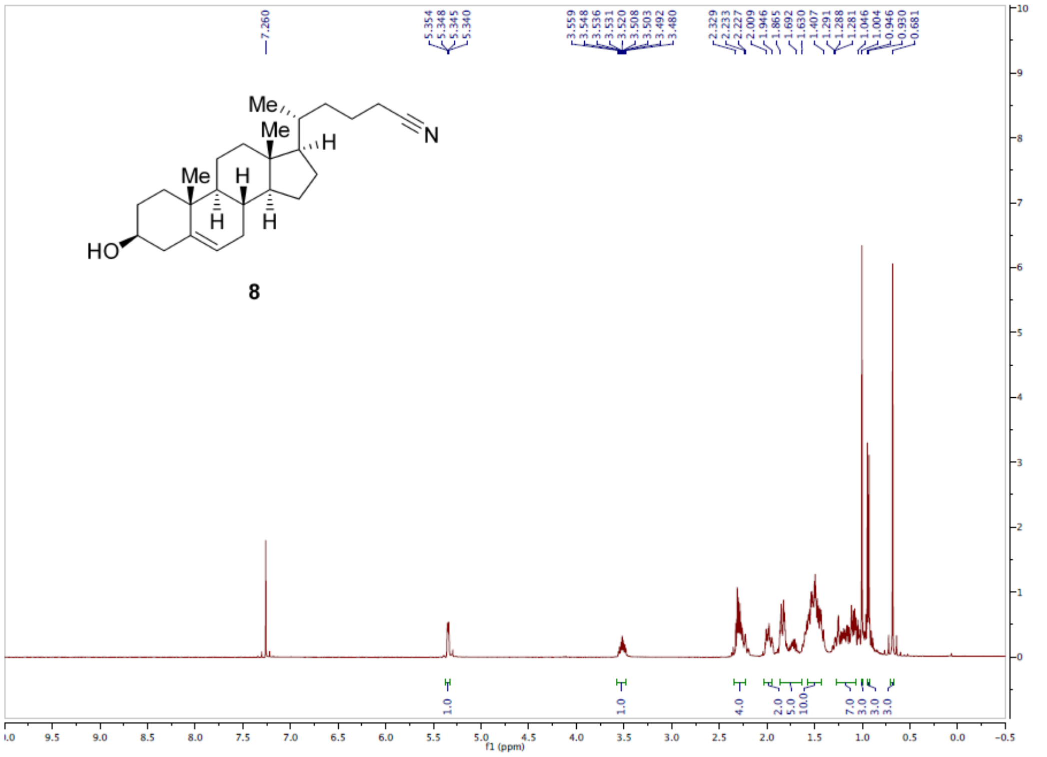

Supplementary Figure 23. ^{1}H NMR of **8** (400 MHz, $CDCl_3$)

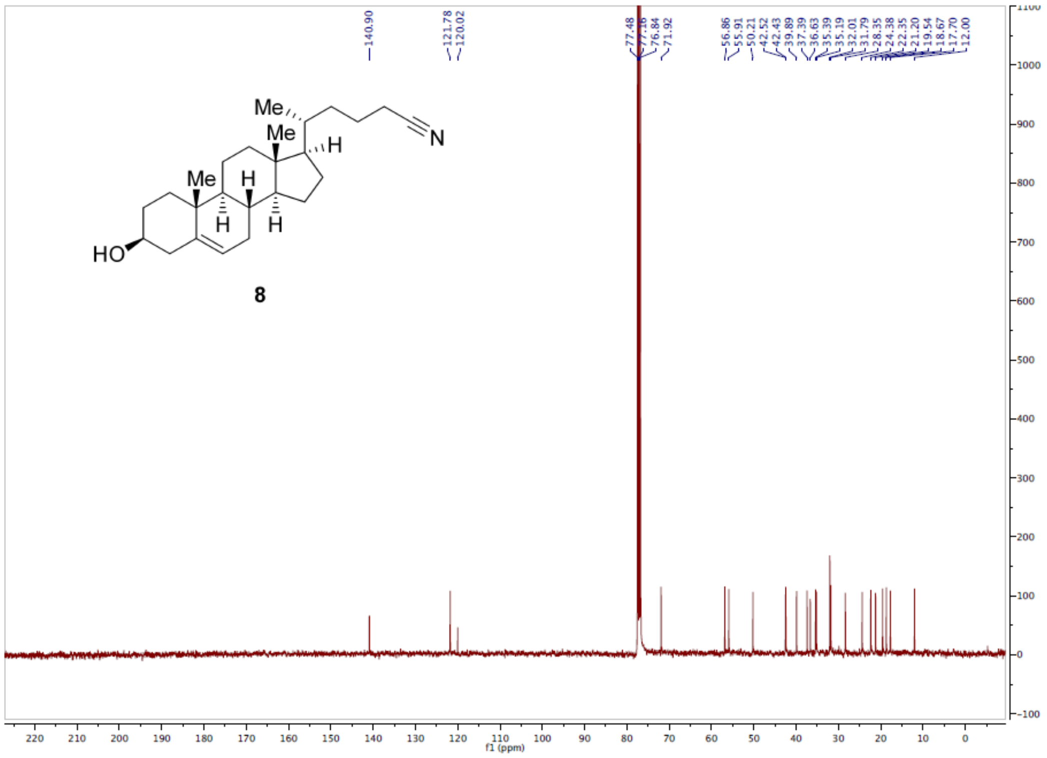

Supplementary Figure 24. ^{13}C NMR of **8** (100 MHz, $CDCl_3$)

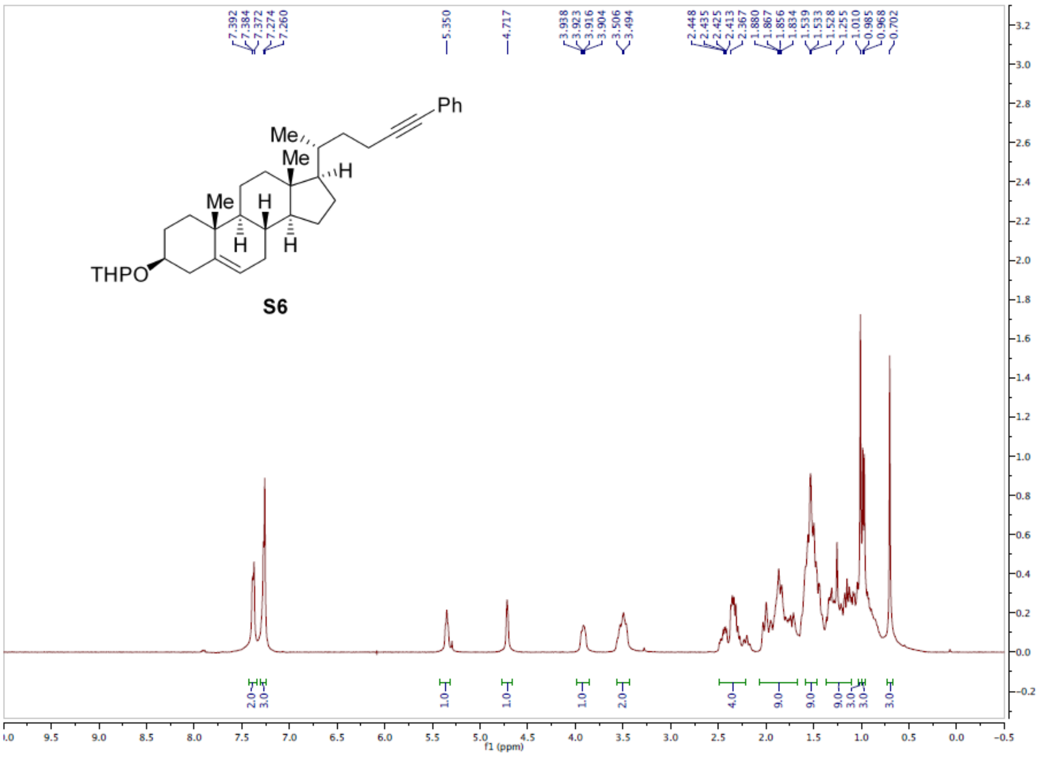

Supplementary Figure 25. ^{1}H NMR of **S6** (400 MHz, $CDCl_3$)

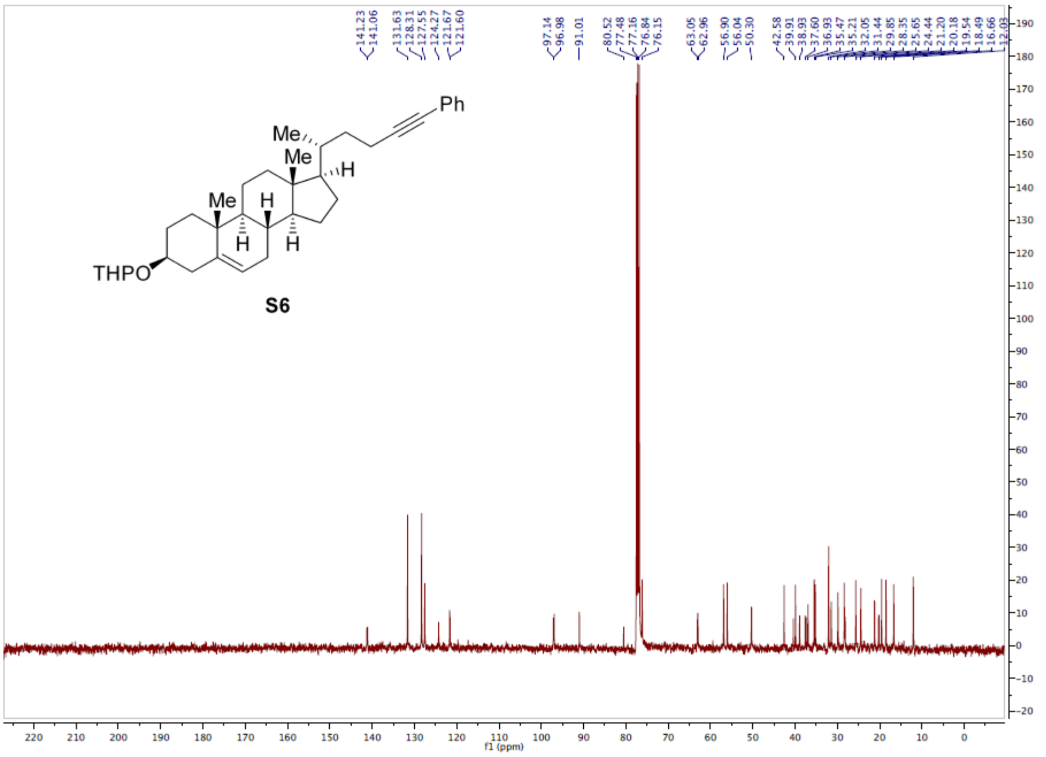

Supplementary Figure 26. ^{13}C NMR of **S6** (100 MHz, $CDCl_3$)

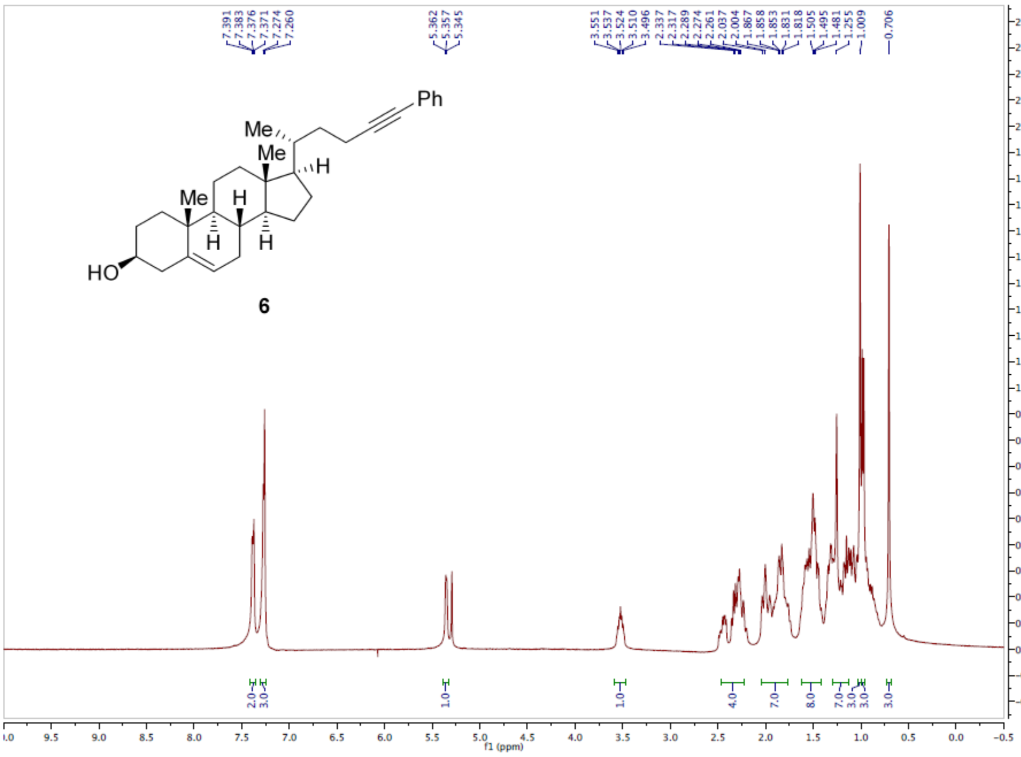

Supplementary Figure 27. ^{1}H NMR of **6** (400 MHz, $CDCl_3$)

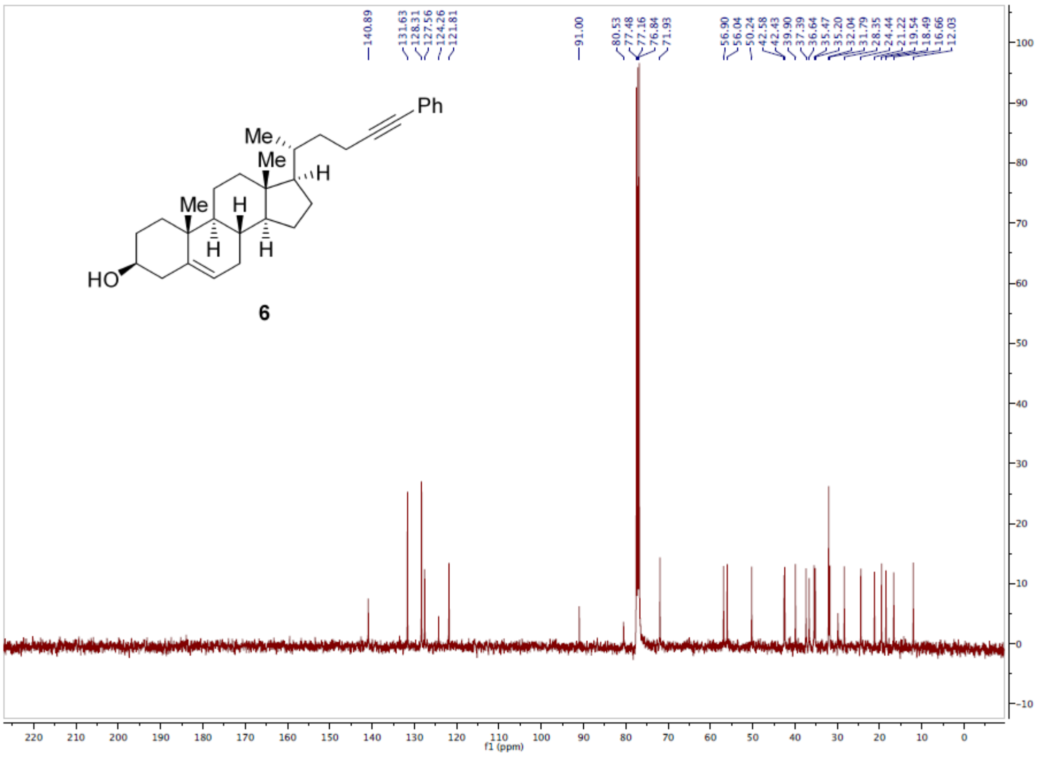

Supplementary Figure 28. ^{13}C NMR of **6** (100 MHz, $CDCl_3$)

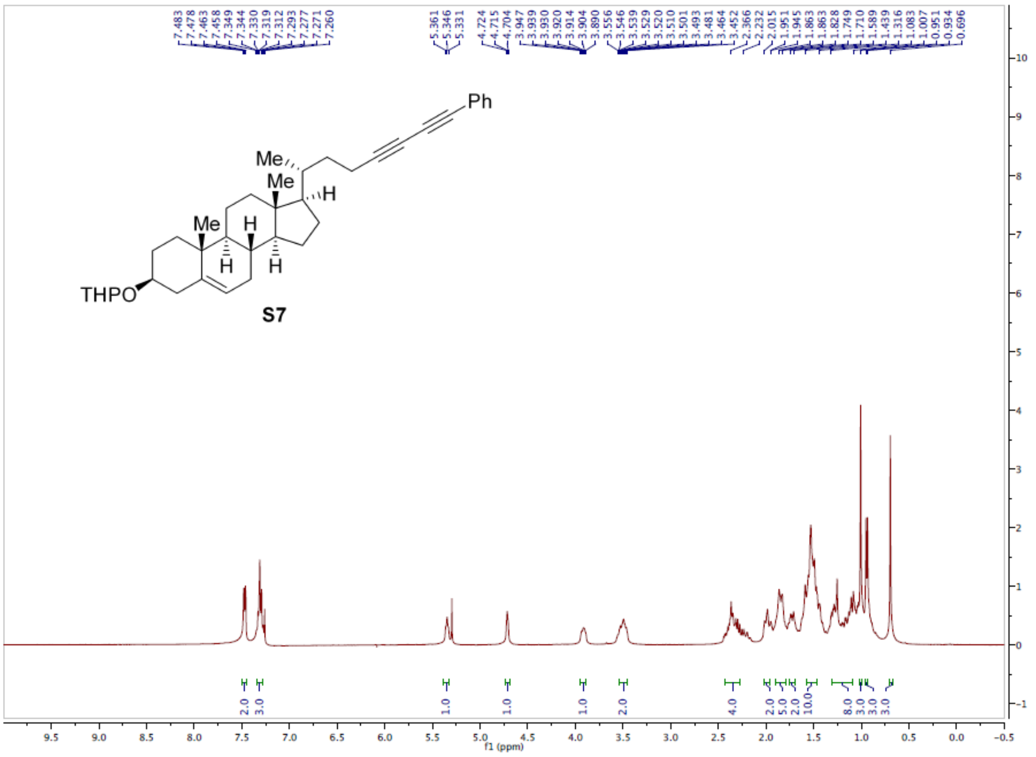

Supplementary Figure 29. ^{1}H NMR of **S7** (400 MHz, $CDCl_3$)

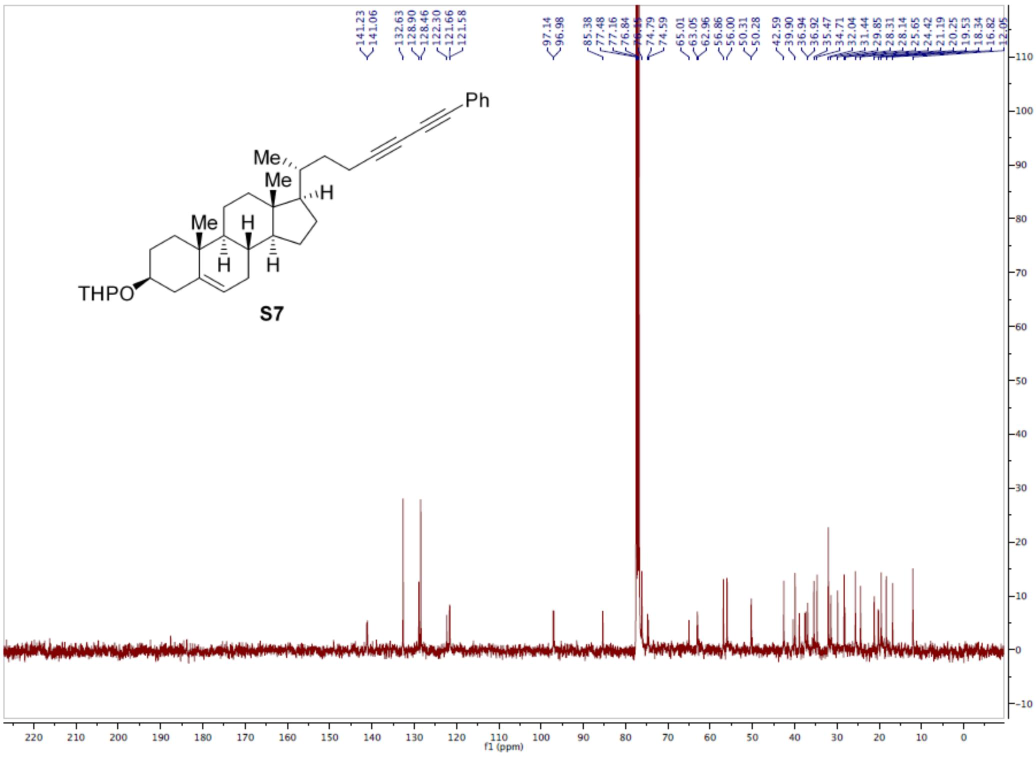

Supplementary Figure 30. ^{13}C NMR of **S7** (100 MHz, $CDCl_3$)

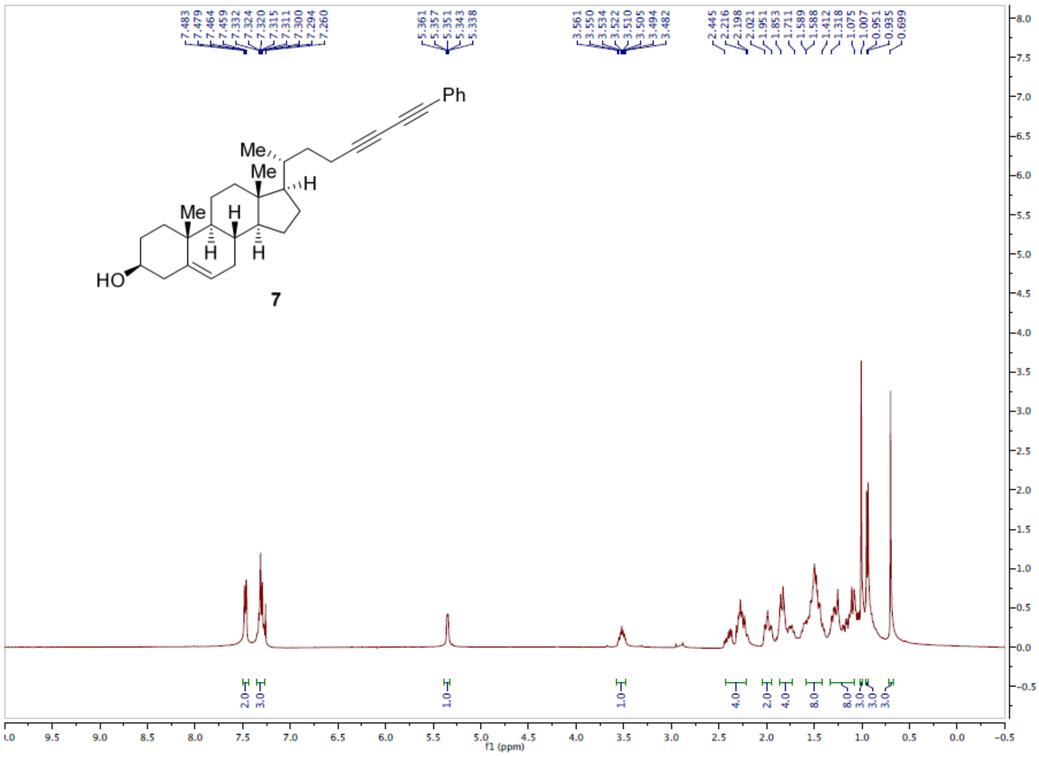

Supplementary Figure 31. ^{1}H NMR of **7** (400 MHz, $CDCl_3$)

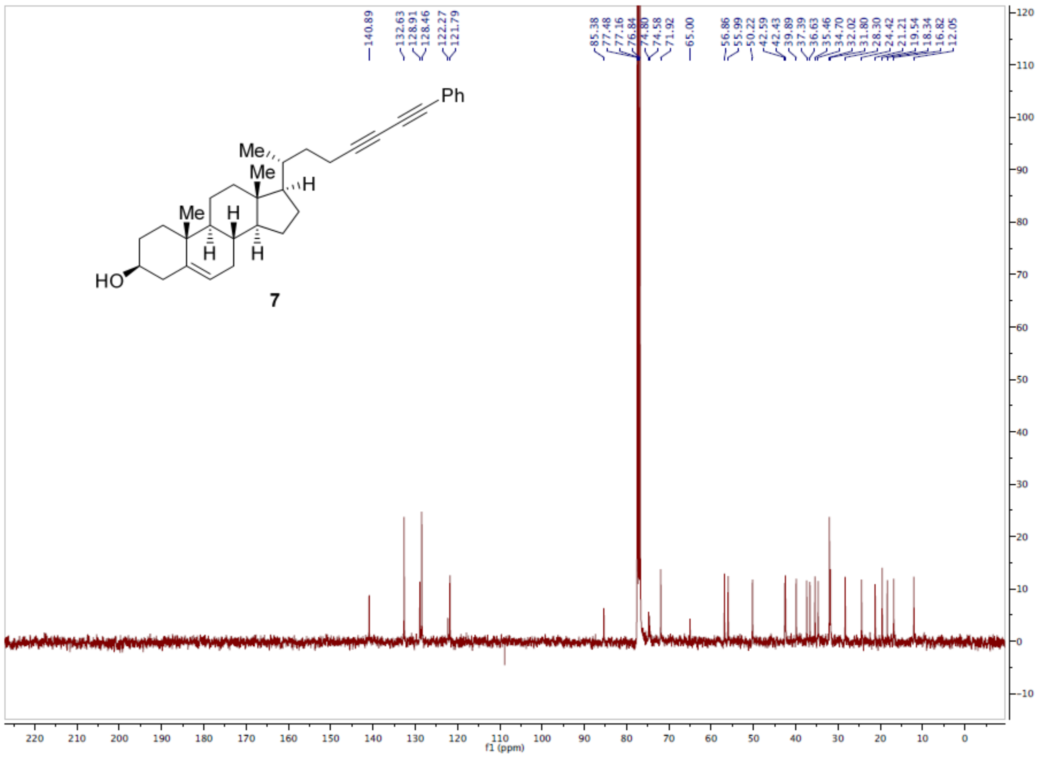

Supplementary Figure 32. ^{13}C NMR of **7** (100 MHz, $CDCl_3$)
